# Investigating the relationship between the comb jellyfish, *Mnemiopsis leidyi*, and the abundance of pathogenic *Vibrio* spp. and harmful algae species in the Maryland Coastal Bays

**DOI:** 10.1128/spectrum.00978-25

**Published:** 2025-12-30

**Authors:** Detbra Rosales, Steve Doctor, John M. Jacobs, Tahirah Johnson, Jennifer L. Wolny, Salina Parveen

**Affiliations:** 1 University of Maryland Eastern Shore 14705, Princess Anne, Maryland, USA; 2 Maryland Department of Natural Resources 11079, Ocean City, Maryland, USA; 3 National Oceanic and Atmospheric Administration, NOS, NCCOS, Cooperative Oxford Laboratory 116032 , Oxford, Maryland, USA; 4 US Food and Drug Administration, Human Foods Program, Office of Laboratory Operations and Applied Science, Office of Applied Microbiology and Technology, Division of Food and Environmental Safety 116043 , College Park, Maryland, USA; Universitetet i Oslo, Oslo, Norway

**Keywords:** *Mnemiopsis leidyi*, *Vibrio*, Maryland Coastal Bays, mid-Atlantic, MPN-PCR, harmful algal blooms

## Abstract

Ctenophores (*Mnemiopsis leidyi*) release large amounts of nutrients and organic matter that stimulate microbial growth and alter planktonic community composition. We investigated the distribution and abundance of *M. leidyi* and its association with pathogenic *Vibrio* and harmful algal bloom (HAB) species at seven sites in the Maryland Coastal Bays (MCBs) between April 2021 and June 2022. Water and *M. leidyi* samples were collected concurrently with temperature, salinity, dissolved oxygen, nutrient, and turbidity data. *M. leidyi* was present in both years at all sites but was most abundant in areas with high turbidity (Isle of Wight and Newport Bays). Both *V. parahaemolyticus* and *V. vulnificus* were present in *M. leidyi* tissue and water samples and had a moderate positive association with turbidity. HAB species composition varied significantly between sites, with distinct populations of the dinoflagellate *Karlodinium veneficum* (Chincoteague Bay) and picoplankton taxa (Newport Bay) noted. A generalized linear model (GLM) revealed that temperature was best at predicting total (*vvhA^+^*) *V. vulnificus* in *M. leidyi* tissues and total dissolved nitrogen (TDN) was best at predicting total (*trh*^+^) *V. parahaemolyticus* in water. There was no strong association between *M. leidyi* abundance and HABs. However, we observed weakly positive associations between *V. vulnificus* in *M. leidyi* tissues and total dinoflagellate biomass, between *M. leidyi* abundance and dinoflagellate biomass, and between *V. parahaemolyticus* in *M. leidyi* tissues and picocyanobacteria biomass. This study provides information on associations between *M. leidyi*, *Vibrio* spp., and HAB species that could aid the development of a *Vibrio* predictive model for the MCBs.

**IMPORTANCE:**

*Vibrio* and algal bloom species naturally occur in marine ecosystems; however, some species of *Vibrio* and algae can be harmful to humans, causing gastroenteritis. *Vibrio*-associated illnesses have been expanding globally in marine environments, and many studies have linked this expansion to increases in water temperatures, nutrients, and plankton blooms. We observed seasonal interactions between *Vibrio* spp., *Mnemiopsis leidyi*, and HABS and found that temperature was the best predictor of total (*vvhA^+^*) *V. vulnificus*, while TDN was the best predictor of total (*trh*^+^) *V. parahaemolyticus*. These findings provide biotic and abiotic factors that managers, researchers, and stakeholders can use in the development of HAB and *Vibrio* spp. mitigation strategies and predictive models for the MCBs.

## INTRODUCTION

Harmful algal bloom (HAB) and *Vibrio* spp. outbreak frequency and organism abundances are increasing in the United States (1–3). A similar pattern has been observed over the past decade in the mid-Atlantic region, particularly within the Chesapeake Bay and the Maryland Coastal Bays (MCBs) (4–6 ). This has led to concerns regarding potential human exposure to pathogenic *Vibrio* spp. that cause gastroenteritis and blood infections and toxin-producing HABs and an increased need for rigorous environmental monitoring and the creation or refinement of organism-specific prediction models (7–9).

The MCBs are composed of numerous shallow, coastal, eutrophic lagoons with restricted water exchange (10, 11). The MCB watershed consists of ~453 km^2^ of mostly agricultural land draining into ~363 km^2^ of bays and tributaries (10, 12). Since the late 1990s, elevated nutrient concentrations have led to recurring HABs, loss of submerged aquatic vegetation, reduced dissolved oxygen concentrations, and overall poorer water quality (6, 13–15). Simultaneously, regional waters have been warming, and blooms of the comb jellyfish, *Mnemiopsis leidyi*, along with outbreaks of pathogenic *Vibrio* species, including *V. parahaemolyticus* and *V. vulnificus*, and the resulting cases of vibriosis, have been on the rise (16–19). However, studies examining the biological interactions of these three groups and corresponding water quality constituents have been limited.

*Vibrio* are naturally occurring Gram-negative marine bacteria that are found in diverse habitats ranging from coastal to open waters (20). They can survive as free-living organisms or attached to organic particles and biofilms (8, 21). *Vibrio* spp. are common components of the MCBs microbial community, with their abundance peaking in the summer (5, 22). Numerous HAB species and *M. leidyi* populations are present year-round in the MCB, and recent studies indicate that their populations are increasing in this region, with peak abundance occurring in late spring to early summer (6, 14, 23, 24). Of particular concern is a regional increase since 2012 of an exceptionally virulent strain of *V. parahaemolyticus* (O3:K6) in Maryland waters, along with a high mortality rate (35%) associated with *V. vulnificus* infections (4).

Temperature and salinity are important drivers for both jellyfish and *Vibrio* populations. As such, these parameters have been used to create an operational forecasting model for blooms of *V. vulnificus* and the Atlantic sea nettle, *Chrysaora quinquecirrha*, in Chesapeake Bay (8, 25, 26). In the MCBs, temperature and salinity also constrain the populations of *M. leidyi* and *Vibrio* spp., but other factors can stimulate their abundance, such as nutrient concentrations (2, 27, 28). Because *Vibrio* spp. can colonize and degrade particulate matter, they play an important role in chemical transformations, including carbon and nutrient cycling (29). Studies (30, 31) suggested that the nutrients that stimulate HABs may also be key to the association between *Vibrio* spp. and phytoplankton. Similarly, *M. leidyi* can also impact nutrient and carbon concentrations by grazing on the various microbes that use and recycle these compounds. Due to their affinity for polysaccharides, *Vibrio* spp. may attach directly to *M. leidyi* and/or attach to the zooplankton consumed by *M. leidyi* (8, 32, 33). Thus, the presence of comb jellyfish may be a driver of *Vibrio* abundance.

While the relationships between *Vibrio* and HAB species (28, 34, 35) and *Vibrio* and jellyfish species (32, 36) have been investigated, the synergy between the three groups has not. Therefore, the objective of this study was to identify the distribution and abundance of these three organismal groups in the MCBs. To do this, *M. leidyi* and HAB species were identified by their morphological characteristics, while *V. parahaemolyticus* and *V. vulnificus* were identified using molecular detection of species-specific and pathogenic genes. Additionally, we examined the presence of *Vibrio* spp. in water and in *M. leidyi* tissues to determine if a relationship between *Vibrio* spp., HABs, and *M. leidyi* existed. Finally, to help refine models used to predict or provide early warnings for the presence of *Vibrio* spp., we examined whether the presence of *M. leidyi* or HABs could be used to predict the presence of *Vibrio* spp. in the MCBs.

## MATERIALS AND METHODS

### Sample collection and phytoplankton enumeration

**Fig 1:**
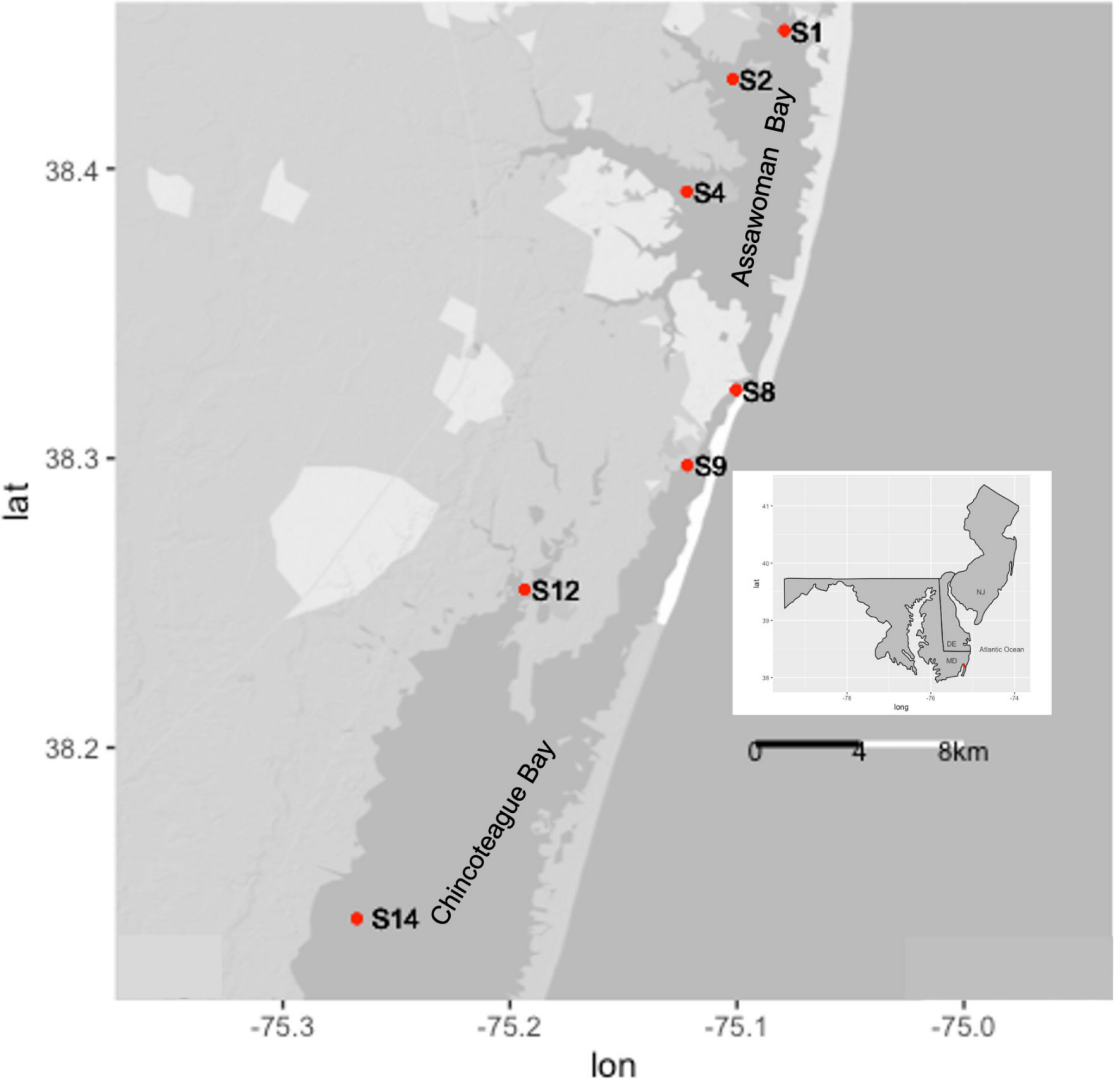
Location of sampling sites in the Maryland Coastal Bays: northern bays (Assawoman [sites S1 and S2] and Isle of Wight [site S4]) and southern bays (Sinepuxent [site S9], Newport [site S12], and Chincoteague [site S14]), divided by Ocean City Inlet (site S8).

A total of 95 water samples, 72 phytoplankton samples, and 43 *M*. *leidyi* samples were collected monthly from seven sites in the MCBs (Fig. 1) from April to October in 2021 and 2022 to examine for *Vibrio* and HAB identification and enumeration. This time period covers spring to autumn and was chosen to capture the variation in *M. leidyi* and *Vibrio* abundance, which often increases in the warmer summer months. The study sites, representing the urbanized northern section of the MCBs (sites: S1 and S2 [Assawoman Bay] and S4 [Isle of Wight Bay]) and the rural southern section of the MCBs (sites: S9 [Sinepuxent Bay], S12 [Newport Bay], and S14 [Chincoteague Bay]) separated by Ocean City Inlet (site S8) were chosen based on differences in water quality and relative abundance of *M. leidyi* and HAB species (10, 14, 37). Temperature, salinity, and dissolved oxygen measurements were collected using a Pro2030 YSI sonde (Yellow Spring Instrument Co., Yellow Springs, OH, USA), and Secchi depths were recorded during each sampling event. Secchi depths were converted to turbidity values (NTU) following Rasmussen et al. (38) with the equation:

$Secchi\;depth\;(ft)=11.123\times x^{-0.637}$

Surface water grab samples for *Vibrio* analysis and water chemistry were collected in 1 L Nalgene wide-mouth bottles from depths of less than 1 m. Phytoplankton samples (150 mL) were collected at 0.5 m depth and preserved immediately with 5% Lugol’s iodine solution (#LC156752, LabChem, Zelienople, PA, USA). *M. leidyi* samples were collected using a semi-balloon trawl net as part of the Maryland Department of Natural Resources trawl survey. Following the methods of Doctor et al. (10), 16 ft semi-balloon otter trawl nets were deployed on the bottom at depths >1.1 m and set for 6 min. After each trawl, 500 mL samples of *M. leidyi* were collected in 1 L zipper plastic bags, stored in a cooler with ice, and processed within 6 h of collection.

Phytoplankton samples, with a focus on HAB species identification and enumeration, were examined using an Olympus CKX41 inverted microscope (Olympus America, Center Valley, PA, USA), following the modified Utermöhl method described by Marshall and Alden (39). The HAB species identified in this study correspond to those reported in Glibert et al. (13), Tango et al. (40), and Wolny et al. (14). All HAB species were identified to the lowest taxon possible using the above-mentioned references in addition to Tomas (41), Steidinger et al. (42), Phlips et al. (43), and Tillmann et al. (44). Cell concentrations were recorded as cells per liter and converted to biomass using the formulae in Hillebrand et al. (45) or Olenina et al. (46). For the purpose of this study, we identified bloom concentrations consistent with regional thresholds proposed by Marshall et al. (47) and Marshall and Egerton (48): dinoflagellate blooms at 10^5^ cells L^−1^, diatom blooms at 10^5^ cells L^−1^, and picoplankton at 10^7^ cells L^−1^, with some species-specific exceptions.

### Quantification of *Vibrio parahaemolyticus* and *V. vulnificus*

Both water and *M. leidyi* samples were processed for most probable number (MPN) analysis in triplicate following the three-tube method of Esteves et al. (49) and Blodgett (50). MPN based on the US Food and Drug Administration’s Bacteriological Analytical Manual (BAM) was used to calculate the abundance of *V. parahaemolyticus* and *V. vulnificus* Blodgett (50). *M. leidyi* samples were poured into a sterile strainer, and the organisms were rinsed with potable tap water. Using sterile forceps, individual *M. leidyi* were picked and added to a sterile blender jar until a total weight of 25 g was reached. An equal amount of phosphate-buffered saline (PBS) was added to the blender jar before blending on high for 90 s. After blending, 1 mL of *M. leidyi* homogenate was inoculated in triplicate into 9 mL of alkaline peptone water (APW) broth and incubated overnight at 35°C. Serial volumes (100 mL, 10 mL, 1 mL, 100 µL, 10 µL, and 1 µL) of undiluted water samples were inoculated in triplicate into 10 mL of APW broth and incubated overnight at static temperature of 35°C. Following incubation, 1 mL of all samples was transferred to microcentrifuge tubes and boiled in a dry bath at 100°C for 10 min and stored at −80°C until real-time PCR analyses were conducted.

*Vibrio* spp. analysis using real-time PCR methods targeted the species-specific gene *tlh* (thermolabile hemolysin) to estimate the abundance of total *V. parahaemolyticus* and the species-specific gene *vvhA* (*Vibrio vulnificus* hemolysin A) to confirm the abundance of total *V. vulnificus* in water. The *tdh^+^* (thermostable direct hemolysin) and *trh^+^* (thermostable related hemolysin) were targeted to determine the presence of virulence genes in *V. parahaemolyticus*. The *vcgC^+^* (virulence correlated gene) was targeted to determine the presence of the virulence gene in *V. vulnificus*. The primers, probes, and internal controls used for these reactions were as described by Panicker and Bej (51), Nordstrom et al. (52), and Baker-Austin et al. (53).

Testing for *tlh*, *tdh^+^*, *trh^+^*, and *vvhA* genes occurred using the National Shellfish Sanitation Program (54) guidelines and an Applied Biosystem 7500 FAST Real-Time PCR system (Thermo Fisher Scientific, Waltham, MA, USA). Reaction concentrations and conditions were as follows: 0.5–0.75 µL of each primer, 0.188–0.5 µL of each probe, 2.5 µL of buffer (Invitrogen, Carlsbad, CA, USA), 2.5 µL MgCl_2_ (Invitrogen, Carlsbad, CA, USA), 0.75 µL dNTP solution (Sigma Aldrich, St. Louis, MO, USA), 11.8 µL of nuclease-free PCR grade water, 0.45 µL platinum Taq polymerase (Invitrogen, Carlsbad, CA, USA), 0.06 µL ROX (Thermo Fisher, Waltham, MA, USA), 2.0 µL of internal amplification control (BioGX, Birmingham, AL, USA), and 2.0 µL of DNA template (boiled MPN culture) for a 25 µL reaction volume with initial denaturation/polymerase activation of 95°C for 60 s, followed by 45 cycles of 95°C for 5 s and an annealing temperature of (57°C for *vvh*A, 59°C for *tlh*, *tdh^+^*, and *trh^+^*) for 45 s (54) .

Testing for the *vcgC^+^* was carried out using an iTaq Universal Supermix (Bio-Rad Laboratories, Hercules, CA, USA), as described by Panicker et al. (55) and Lane et al. (56), using a Bio-Rad CFX96 RT PCR system with an initial denaturation/polymerase activation of 95°C for 180 s, followed by 45 cycles of 95°C for 5 s and an annealing temperature of 60°C for 45 s.

### Nutrient analysis

For nutrient analyses, 120 mL water samples were filtered through a Sterivex cartridge (0.22 µm, Millipore, Merck, Burlington, MA, USA) using a 50 mL Luer-Lock syringe (BD Medical, Franklin Lakes, NJ, USA). Filtrates were stored at −80°C until analysis. Concentrations of total dissolved nitrogen (TDN), total dissolved phosphorus (TDP), (nitrate/nitrite, (NO_3_:NO_2_), nitrite (NO_2_^-^), ammonium (NH_4_^+^), and ortho-phosphate (PO_4_^3-^) were analyzed by the Chesapeake Biological Laboratory (Solomons, MD, USA) using a Technicon Auto Analyzer II (SEAL Analytical Inc., Mequon, WI, USA) and NAP software following the methods of the US Environmental Protection Agency, as modified by the Chesapeake Bay Program (57, 58).

### Statistical analysis

All statistical analyses were conducted using RStudio version 3.3.0 (RStudio, 2015). *Vibrio* spp. and HAB species enumeration data were log-transformed to normalize the data distribution. Site maps were created with Ggmaps, integrating base maps from Google Maps using RStudio (V 3.3.0) (59). The relationships between *M. leidyi*, HABs, *Vibrio* spp., nutrients, biomass, and environmental parameters were evaluated using a one-way ANOVA in RStudio. The ggplot2 package in RStudio was used to generate a visual representation of the distribution of *M. leidyi*, HABs, *Vibrio* spp., and environmental parameters (V3.3.0) (60). A Spearman correlation test and a Benjamini and Hochberg *P* value adjustment were used to assess the association between *M. leidyi*, *Vibrio* spp., nutrients, HABs, and environmental parameters. Seasons in this study are defined as spring (April–June) and summer (July–October) based on regional climate/weather patterns and phytoplankton and fish populations (14, 61, 62). Raphidophyte data were removed from seasonal correlation analysis because they were not frequently present in the study area. Initially, all picoplankton-sized taxa (those >3 µm) were categorized together. However, due to different environmental preferences and ecosystem disruptive modes (43, 63, 64), picoplankton-sized taxa were analyzed separately as *Aureococcus anophagefferens*, Pedinophyceae, and picocyanobacteria. Correlograms (package Corrgram in RStudio) were used to produce a visual representation of the relationship between environmental factors and to help identify what variables to use when modeling.  

Generalized linear models (GLMs) were used to describe the relatedness between *Vibrio* spp. and environmental variables. *Vibrio* spp. markers in environmental samples were used as the response variables, and environmental parameters were the explanatory variables used in each model. Combinations of each model are found in Tables 1 and 2. The MuMIn package in RStudio was used to calculate AICc criteria to rank the models (65). AICc is a statistical method used for model selection, helping to identify the model that best fits the data (66). The response variable was *vvhA*^+^ *V. vulnificus* and *trh V. parahaemolyticus* in *M. leidyi*, and the fixed variables were environmental parameters, including nutrients. The models included both abiotic and biotic variables. Additionally, the fitness of the model was assessed in R studio using base R and the sensitivity package to assess the reliability of the models and the extent to which model predictions are influenced by changes in input variables (67). Specifically, we calculated the estimated coefficients and the percent change in model predictions resulting from a 10% increase in each predictor variable.

**TABLE 1:** Model comparison of general linear models with *Vibrio* spp. markers in *Mnemiopsis leidyi* tissues as the response variable

Model no	Explanatory variables	AICc^*a*^	Delta	Weight^*b*^	RMSE^*c*^
M7	*vvhA* ~ Temp	31.6	0.00	0.679	0.402
M10	*vvhA* ~ Temp + Turb + Sal + DO	34.5	2.86	0.163	0.402
Null model	*vvhA* ~ 1	35.4	3.75	0.104	0.455
M9	*vvhA* ~ Temp + Sal + DO	37.4	5.84	0.037	0.399
M8	*vvhA* ~ Temp + Sal	38.9	7.30	0.018	0.382
Model no	Explanatory variables	AICc^*a*^	Delta	Weight^*b*^	RMSE^*c*^
M6	*Tlh* ~ Turb + Temp	98.2	0.00	0.384	1.51
M7	*Tlh* ~ Turb	99.6	1.47	0.184	1.66
Null	*Tlh ~ 1*	99.7	1.50	0.182	1.75
M3	*Tlh ~* Turb + Temp + Sal + DO	101.1	2.96	0.088	1.40
M5	*Tlh* ~ Turb + Sal	101.2	3.07	0.083	1.61
M4	*Tlh ~* Turb + Temp + Sal	101.3	3.14	0.080	1.51
Model no	Explanatory variables	AICc^*a*^	Delta	Weight^*b*^	RMSE^*c*^
M6	*Trh* ~ Turb + Temp	80.6	0.00	0.357	1.05
Null	*Trh ~ 1*	80.8	0.22	0.320	1.18
M7	*Trh* ~ Turb	82.3	1.73	0.150	1.16
M4	*Trh ~* Turb + Temp + Sal	83.4	2.84	0.086	1.04
M3	*Trh ~* Turb + Temp + Sal + DO	84.6	3.99	0.049	1.00
M5	*Trh* ~ Turb + Sal	85.0	4.47	0.038	1.15

^*a*^ AICc, Akaike’s information criterion. A lower AICc indicates a better model.

^*b*^ wt, weight.

^*c*^ RMSE, root-mean-square deviation.

**TABLE 2:** Model comparison of general linear models with *Vibrio* spp. markers in environmental samples as the response variable

Model no	Explanatory variables	AICc^*a*^	Delta	Weight^*b*^	RMSE^*c*^
M7	*vvhA* ~ Turb	0.1	0.00	0.633	0.208
M5	*vvhA* ~ Turb + Sal	2.6	2.75	0.160	0.207
M6	*vvhA* ~ Turb + Temp	2.7	2.86	0.152	0.207
M4	*vvhA ~* Turb + Temp + Sal	5.8	5.97	0.032	0.207
Null	*vvhA ~* 1	7.1	7.23	0.017	0.255
M3	*vvhA ~* Turb + Temp + Sal + DO	9.3	9.46	0.006	0.207
Model no	Explanatory variables	AICc^*a*^	Delta	Weight^*b*^	RMSE^*c*^
M7	*Tlh* ~ Turb	53.6	0.00	0.516	0.636
M5	*Tlh* ~ Turb + Sal	55.6	2.01	0.189	0.624
M6	*Tlh* ~ Turb + Temp	55.8	2.24	0.168	0.627
M4	*Tlh ~* Turb + Temp + Sal	56.8	3.19	0.105	0.598
M3	*Tlh ~* Turb + Temp + Sal + DO	60.4	6.80	0.017	0.598
Null	*Tlh ~* 1	62.8	9.20	0.005	0.814
Model no	Explanatory variables	AICc^*a*^	Delta	Weight^*b*^	RMSE^*c*^
M7	*Trh* ~ Turb	19.3	0.00	0.472	0.311
M5	*Trh* ~ Turb + Sal	20.0	0.76	0.322	0.297
M6	*Trh* ~ Turb + Temp	22.1	2.86	0.113	0.311
M4	*Trh ~* Turb + Temp + Sal	23.0	3.74	0.073	0.296
M3	*Trh ~* Turb + Temp + Sal + DO	26.2	6.94	0.015	0.293
Null	*Trh ~* 1	28.0	8.77	0.006	0.394
Model no	Explanatory variables	AICc^*a*^	Delta	Weight^*b*^	RMSE*^c^*
M7	*Tdh* ~ Turb	70.6	0.00	0.516	0.048
M5	*Tdh* ~ Turb + Sal	69.7	0.90	0.189	0.046
M6	*Tdh* ~ Turb + Temp	67.8	2.84	0.168	0.048
M4	*Tdh ~* Turb + Temp + Sal	67.6	3.06	0.105	0.045
M3	*Tdh ~* Turb + Temp + Sal + DO	64.1	6.55	0.017	0.045
Null	*Tdh ~* 1	62.6	8.03	0.005	0.060

^*a*^ AICc, Akaike’s information criterion. A lower AICc indicates a better model.

^*b*^ wt, weight.

^*c*^ RMSE, root-mean-square deviation.

## RESULTS

### Comparison of environmental parameters

Abiotic parameters were measured at all seven sites in MCBs (Fig. 1) to establish the relative water quality between sites. The averages and ranges of water temperature, salinities, dissolved oxygen, turbidity, and nutrient concentrations are shown in Tables 3 and 4. A one-way ANOVA showed no significant difference in temperature between sites (Table S1). Salinities ranged from 19 to 36 PSU, with higher salinity values recorded near sites S14, S8, and S4 due to the influence of Ocean City Inlet and the lowest values recorded at S12. Overall, the salinities were greater in 2022 than in 2021. There was a significant difference in salinity between sites and years with a *P* < 0.05 (Table 3 and 4; Table S1).

**TABLE 3:** Average distribution of *Mnemiopsis leidyi* and environmental parameters in the Maryland Coastal Bays from 2021 to 2022^*h*^

	Maryland Coastal Bays	
2021–2022	Average 2021	Average 2022
Dinoflagellate^*a*^	1.24 × 10**^4^**	5.80 × 10**^2^**
Picocyanobacteria^*a*^	0	0.16 × 10**^1^**
Diatoms^*a*^	5.40 × 10**^2^**	2.90 × 10**^−1^**
*M. leidyi*^*b*^	9.47	7.15
Temperature ^*c*^	22.10	20.70
Salinity ^*d*^	27.00	29.30
Dissolved oxygen^*e*^	6.55	6.50
Turbidity ^*f*^	10.50	8.10
2021–2022	Average 2021	Average 2022
NH_4_^+ *g*^	0.028	0.036
NO_3_^-^:NO_2_^- *g*^	0.018	0.021
TDN ^*g*^	0.401	0.399
PO_4_^3-*g*^	0.008	0.081
TDP^*g*^	0.022	0.092

^*a*^ Biomass μg Carbon L^−1^.

^*b*^ Density mL^−1^.

^*c*^ °C.

^*d*^ psu.

^*e*^ mg L^−1^.

^*f*^ NTU.

^*g*^ μM.

^*h*^ For 2021 sample size (Water = 49 and *M. leidyi *= 30). For 2022 sample size ( Water = 46 and *M. leidyi* = 13).

**TABLE 4:** Average distribution of *Mnemiopsis leidyi* and abiotic parameters in the Maryland Coastal Bays from 2021 to 2022^*g*^^,^^*h*^

Maryland coastal bays								
Values by location								
Sites	S1	S2	S4	S8	S9	S12	S14	*P* value
*M. leidyi* ^*a*^	6.23	4.54	13.10	4.79	8.36	12.10	9.29	**5.1 × 10^−3^**
Temperature^*b*^	22.10	22.30	21.30	19.4	20.80	22.20	22.20	8.1 × 10^−1^
Salinity^*c*^	26.80	27.10	27.60	30.5	30.40	24.40	29.40	**3.29 × 10^−4^**
DO^*d*^	6.12	6.26	6.82	6.94	6.81	6.16	6.47	5.6 × 10^−1^
Turbidity^*e*^	7.6	11.2	11.8	6.5	6.3	16.6	6.3	**1.3 × 10^−4^**
NH_4_^+^^*f*^	0.08	0.06	0.02	0.01	0.02	0.02	0.01	**7.6 × 10^−6^**
NO_3_^-^:NO_2_^-^^*f*^	0.03	0.03	0.01	0.02	0.05	0.01	0.01	**3.3 × 10^−6^**
TDN^*f*^	0.58	0.58	0.04	0.21	0.25	0.54	0.31	**5.9 × 10^−11^**
PO_4_^3-^^*f*^	0.04	0.03	0.01	0.04	0.05	0.06	0.07	9.1 × 10^−1^
TDP^*f*^	0.06	0.06	0.04	0.50	0.03	0.04	0.11	7.6 × 10^−1^

^*a*^ Density mL^−1^.

^*b*^ °C.

^*c*^ psu.

^*d*^ mg L^−1.^

^*e*^ NTU.

^*f*^ μM.

^*g*^ *P *values were determined by a one-way ANOVA.

^*h*^ The bold are highlighting *P *values less than 0.05 and there is significant difference between years.

Dissolved oxygen levels were similar between sites, averaging between 6.2 and 6.8 mg mL^−1^. Turbidity ranged from 0.001 to 34.10 NTU. The average turbidity was highest at sites S12 (16.6 NTU) and S4 (11.8 NTU). A one-way ANOVA showed no significant difference between dissolved oxygen levels, but there was a difference in turbidity values between sites with a *P* < 0.05 (Tables 3 and 4; Table S1).

Nutrient concentrations are given in Tables 3 and 4. One-way ANOVA showed that there were no significant differences in TDN (0.11–1.10 µM), NO_3_^-^:NO_2_ (0.01–0.48 µM), and NH_4_^+^ (0.01–0.29 µM) concentrations between years. However, there were significant differences in TDN, NO_3_^-^:NO_2_, and NH_4_^+^ concentrations between sites with a *P* < 0.05. The average TDN concentrations were highest at sites S1 and S2 (both 0.58 µM) and at site S12 (0.54 µM). The average NO_3_^-^:NO_2_ concentrations were highest at sites S1 and S2 (both 0.03 µM) and at site S9 (0.05 µM). The average NH_4_^+^ concentrations were highest at sites S1 (0.08 µM) and S2 (0.06 µM). There were also significant differences in PO_4-_ (0–0.62 µM) and TDP (0.01–0.83 µM) concentrations between years with a *P* < 0.05. The average PO_4-_ and TDP concentrations were higher in 2022 compared to 2021, and there were no significant differences in PO_4-_ and TDP concentrations between sites (Tables 3 and 4; Table S1).

### HAB species distribution in the MCBs

Site distribution of the HAB species identified in the MCBs is shown in Fig. S1a. A HAB species list, with known harmful effects, is provided in Table S2. Northern MCB sites (S1, S2, and S4) had consistent populations of the bloom-forming dinoflagellates *Levanderina fissa*, *Akashiwo sanguinea*, and *Gymnodinium aureolum*. The potentially toxic or harmful diatoms, *Pseudo-nitzschia* spp. and *Proboscia alata*, were most abundant at sites near Ocean City Inlet (S8 and S9). The phytoplankton community at the Newport Bay site (S12) had a higher percentage of picoplankton-sized cells (2–3 µm), including picocyanobacteria, an unidentified Pedinophyceae species, and the brown tide organism *Aureococcus anophagefferens*, compared to other HAB species and compared to other study sites (Fig. S1). The ichthyotoxic dinoflagellate, *Karlodinium veneficum*, was the most abundant HAB species found in Chincoteague Bay (S14). The bloom-forming dinoflagellate *Prorocentrum minimum* and the ciliate *Mesodinium rubrum*, which plays a vital role in the life cycle of dinoflagellates in the genus *Dinophysis* and can serve as a predictive indicator for *Dinophysis* blooms (68), were found throughout the MCBs during this study.

**Fig 2:**
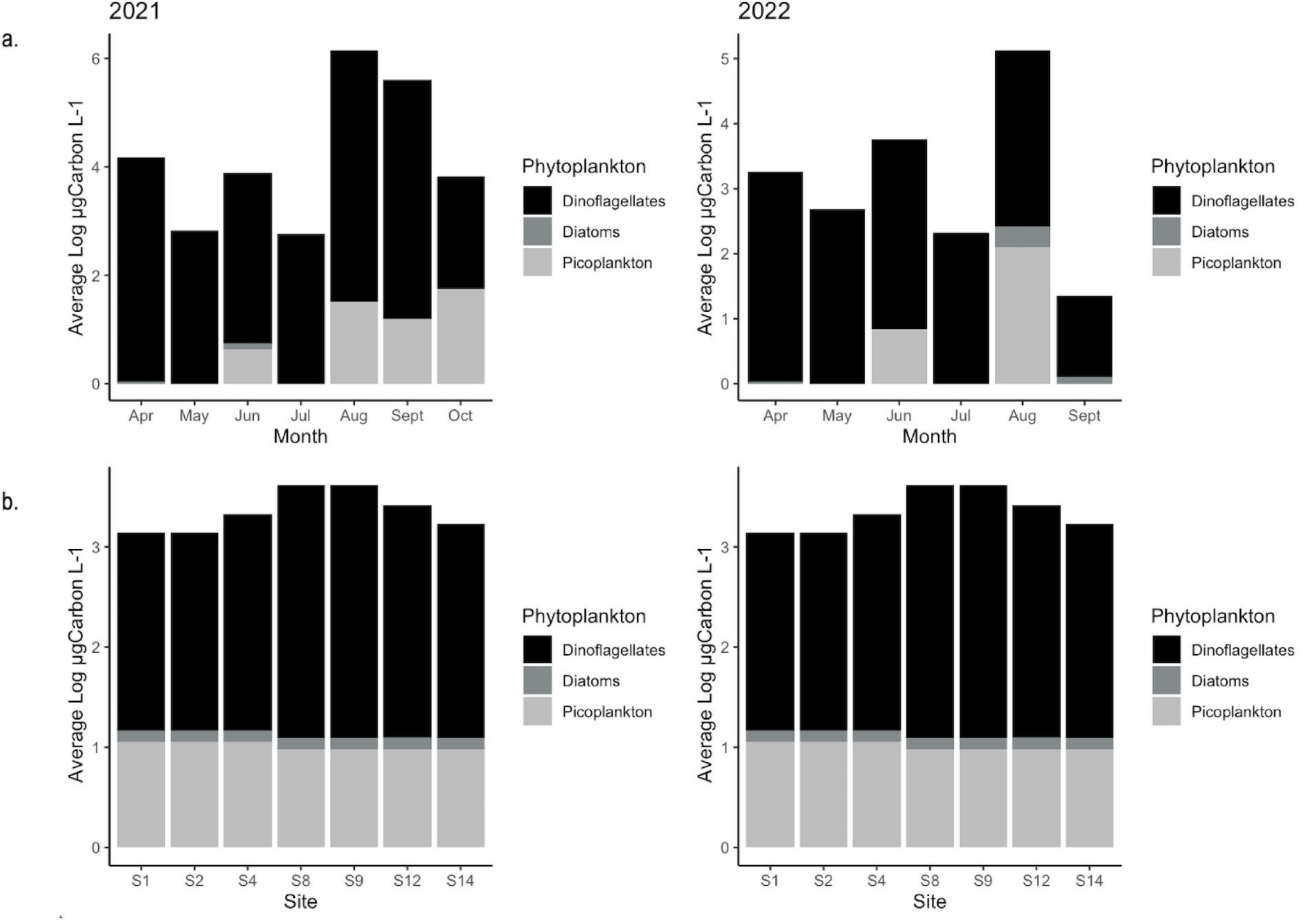
Average biomass of major phytoplankton groups observed in the Maryland Coastal Bays in 2021–2022. (**a**) Average biomass by month. (**b**) Average biomass by site.

One-way ANOVA suggested that there was no significant difference in HAB concentrations between sites. However, there was a significant difference in picoplankton, diatom, and dinoflagellate biomass between years and months with a *P* value < 0.05 (Fig. 2 and Table S1). Total picocyanobacteria biomass was 0.73 × 10^1^ μgC L^−1^ (maximum observed in September 2022), 0.18 × 10^1^ μgC L^−1^ for diatoms (maximum observed in September 2022), and 7.2 × 10^3^ μgC L^−1^ for dinoflagellates (maximum observed in September 2021).

### *Mnemiopsis leidyi* distribution in MCBs

**Fig 3:**
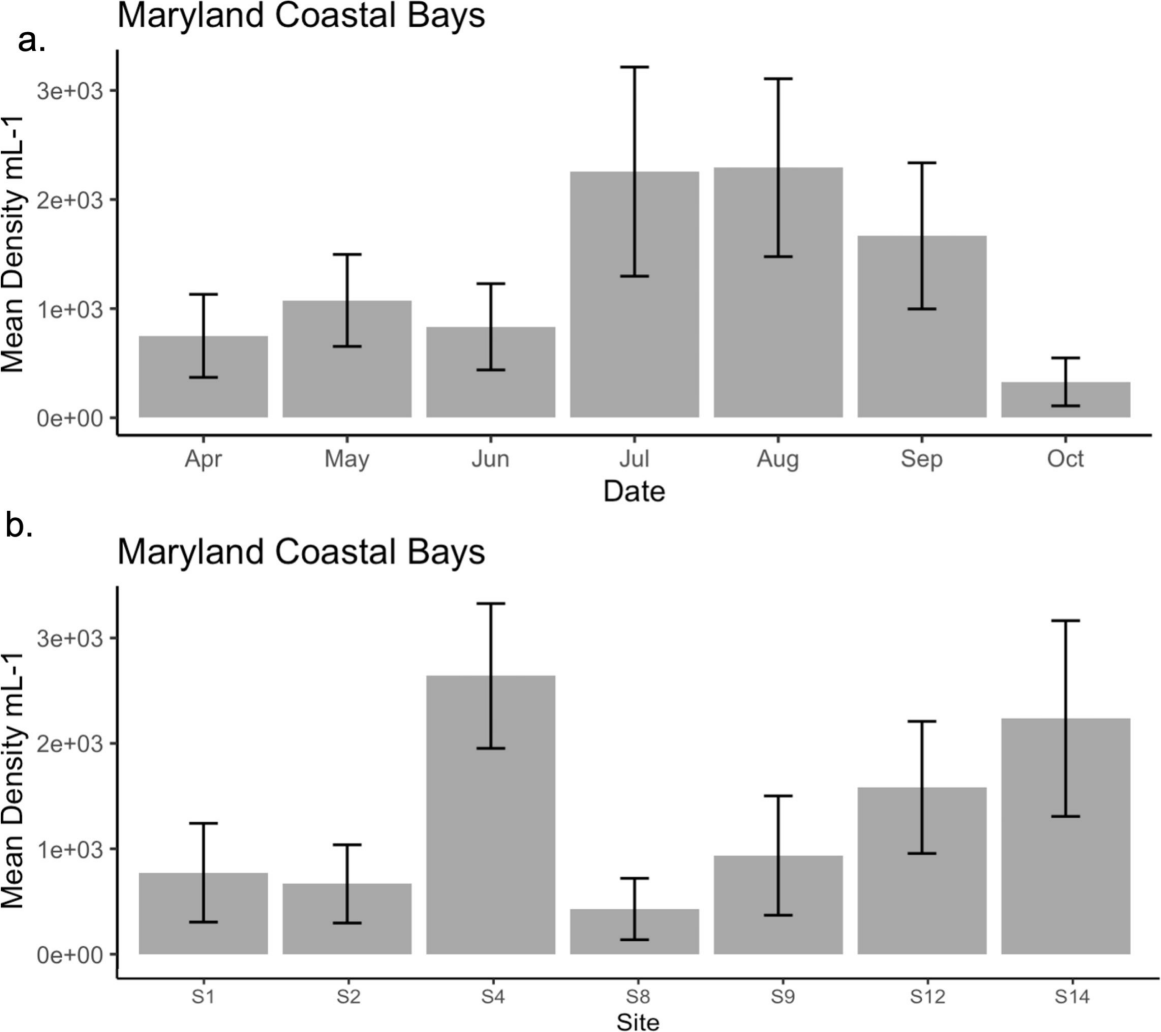
Distribution of *Mnemiopsis leidyi* in the Maryland Coastal Bays from 2021 to 2022. (**a**) Mean density by month, *n* = 43. (**b**) Mean density by site, *n* = 43. The bar plots summarize mean per mL concentrations of *M. leidyi* with standard errors.

Seasonal and temporal variations in *M. leidyi* abundances are shown in Fig. 3. *M. leidyi* was most abundant from June to August, and the concentrations were highest at sites S4 and S14. A one-way ANOVA revealed a significant difference in abundance between sites with a *P* < 0.05 (Table 4). However, there was no significant difference in *M. leidyi* abundance between years (Table S1).

### *Vibrio parahaemolyticus* prevalence in water and *Mnemiopsis leidyi* tissues

The concentrations and detection percentage of *V. parahaemolyticus* in water samples and *M. leidyi* tissues using the species-specific and pathogenic genes are listed in Table 5. The thermolabile hemolysin (*tlh*) *V. parahaemolyticus* gene was detected in 70% of water samples, and the concentrations ranged from 0.01 to 3.72 log MPN mL^−1^. In homogenized tissue samples of *M. leidyi*, the *tlh V. parahaemolyticus* gene was detected in 56% samples, and the concentrations ranged from 0.44 to 4.90 log MPN g^−1^. The pathogenic thermostable direct hemolysin positive (*tdh*^+^) *V. parahaemolyticus* gene was detected in 15% of water samples and 5% of homogenized *M. leidyi* tissue samples. Concentrations of the *tdh*^+^ *V. parahaemolyticus* gene in water ranged from 0.01 to 0.28 log MPN mL^−1^ and from 0.28 to 1.85 log MPN g^−1^ in homogenized *M. leidyi* tissue. The pathogenic thermostable related hemolysin positive (*trh*^+^) *V. parahaemolyticus* gene was detected in 16% of water samples and 28% of homogenized *M. leidyi* tissue samples. Concentrations of the *trh*^+^ *V. parahaemolyticus* gene in water ranged from 0.01 to 1.87 log MPN mL^−1^ and from 0.49 to 4.06 log MPN g^−1^ in homogenized *M. leidyi* tissues (Table 5). There was a significant difference in total *tlh*^+^ *V. parahaemolyticus* gene found in water samples between years, with more *V. parahaemolyticus* detected in 2021 than 2022 (*P* < 0.05; Table 6).

**TABLE 5:** Distribution of *Vibrio* spp. in the Maryland Coastal Bays

	Water and *M. leidyi*					
	2021–2022					
Variable		Number positive/total	Percent positive	Min	Max	Average
*V. parahaemolyticus* (*tlh*^+^)^*a*^		64/92	70%	0.01	3.72	0.40
*V. parahaemolyticus* (*tlh*^+^)^*b*^		22/39	56%	0.44	4.90	1.19
*V. parahaemolyticus* (*tdh*^+^)^*a*^		14/92	15%	0.01	0.28	0.01
*V. parahaemolyticus* (*tdh*^+^)^*b*^		2/39	0.05%	0.28	1.85	0.04
*V. parahaemolyticus* (*trh*^+^)^*a*^		15/92	16%	0.01	1.87	0.06
*V. parahaemolyticus* (*trh*^+^)^*b*^		11/39	28%	0.49	4.06	0.36
*V. vulnificus* (*vvhA*^+^)^*a*^		18/92	20%	0.02	1.40	0.08
*V. vulnificus* (*vvhA*^+^)^*b*^		13/39	33%	0.05	2.11	0.16
*V. vulnificus* (*vcgC*^+^)^*a*^		1/92	1%	0.00	0.03	3.26 × 10^−4^
*V. vulnificus* (*vcgC*^+^)^*b*^		3/39	8%	0.30	2.04	0.010

^*a*^ log MPN mL^−1^.

^*b*^ log MPN g^−1^.

**TABLE 6:** Average distribution of *Vibrio* spp. by year in the Maryland Coastal Bays^*c*^^,^^*d*^

Variable	Avg by year		
	2021	2022	*P* value
*V. parahaemolyticus* (*tlh*+)*^a^*	1.50	0.50	**1.1 × 10^−2^**
*V. parahaemolyticus* (*tlh*+)^*b*^	0.40	0.39	4.9 × 10^−1^
*V. parahaemolyticus* (*tdh*+)^*a*^	0.01	0.02	3.1 × 10^−1^
*V. parahaemolyticus* (*tdh*+)^*b*^	0.01	0.13	6.3 × 10^−1^
*V. parahaemolyticus* (*trh*+)^*a*^	0.07	0.05	7.2 × 10^−1^
*V. parahaemolyticus* (*trh*+)^*b*^	0.46	0.11	5.7 × 10^−1^
*V. vulnificus* (*vvhA*+)^*a*^	0.12	0.02	**8.3 × 10^−3^**
*V. vulnificus* (*vvhA*+)^*b*^	0.18	0.08	3.1 × 10^−1^
*V. vulnificus* (*vcgC*+)^*a*^	6.00 **×** 10^−4^	0.00	9.2 × 10^−1^
*V. vulnificus* (*vcgC*+)^*b*^	0.12	0.03	4.4 × 10^−1^

^*a*^ log MPN mL^−1^.

^*b*^ log MPNg^−1^.

^*c*^ *P *values were determined by a one-way ANOVA. For 2021 sample size (Water = 49 and *M. leidyi* = 30). For 2022 sample size ( Water = 46 and *M. leidyi *= 13).

^*d*^ The bold are highlighting *P* values less than 0.05 and there is significant difference between years.

**Fig 4:**
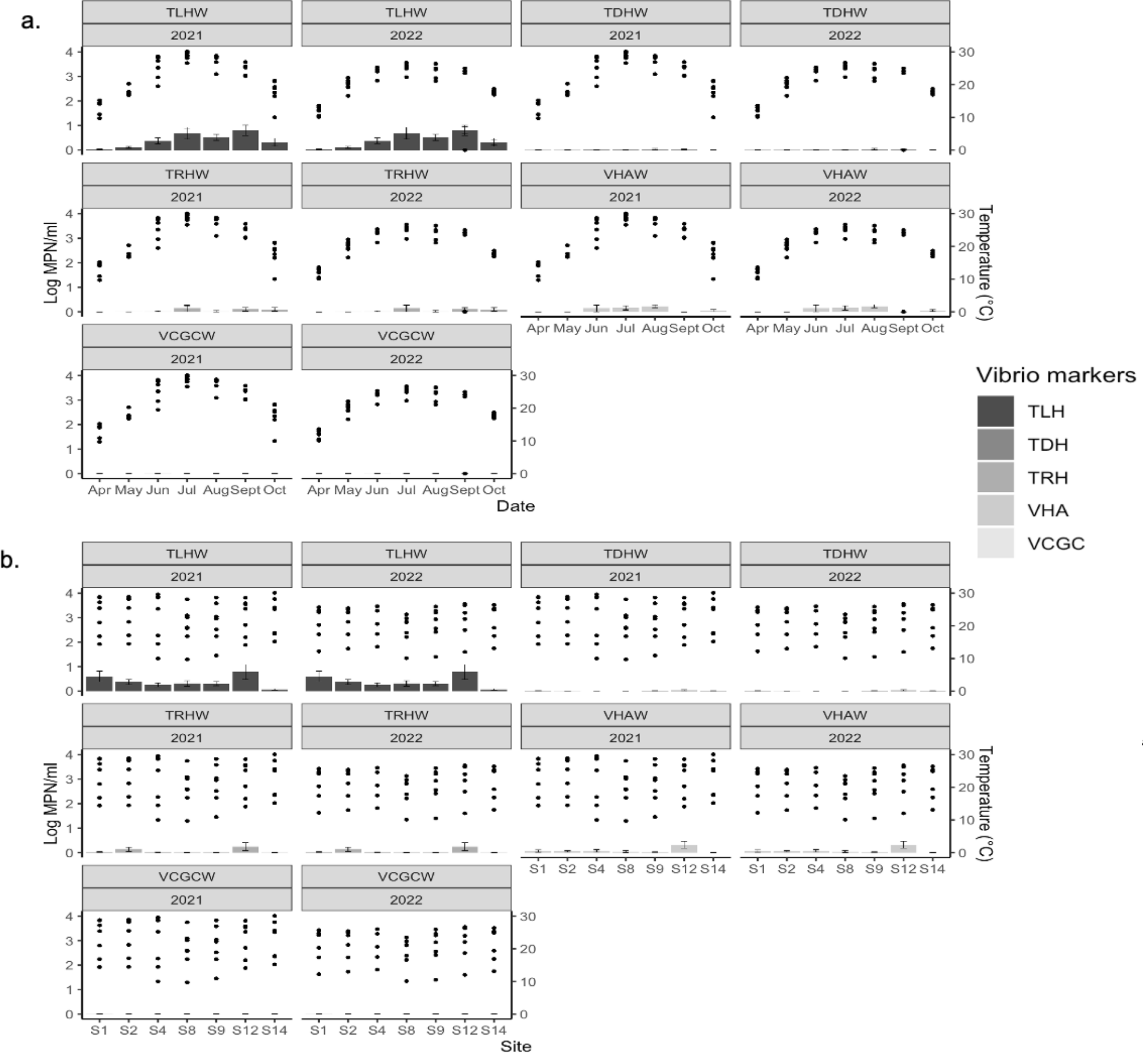
Temperature distribution overlaid with *Vibrio parahaemolyticus* (*tlh*), *V. vulnificus* (*vvhA*), pathogenic *V. parahaemolyticus* (*tdh*^+^ and *trh*^+^), and pathogenic *V. vulnificus* (vcgC^+^) occurrence data in the Maryland Coastal Bays for 2021–2022. (**a**) Means and standard errors for *Vibrio* genes within months, *n* = 95. (**b**) Means and standard errors for *Vibrio* genes within sites, *n* = 95.

The *tlh V. parahaemolyticus* gene was detected in water samples monthly, but the highest concentrations were observed from July to September (Fig. 4a). The *tlh V. parahaemolyticus* gene was detected at all seven sites, but the highest concentrations in water samples were detected at sites S1 and S12 (Fig. 4b). The *tdh*^+^ *V. parahaemolyticus* gene was detected at low concentrations from June to September (*tdh*^+^) at sites S1, S9, and S12, and the *trh*^+^ *V. parahaemolyticus* gene was detected at low concentrations from June to October at sites S1, S2, S4, and S12 (Fig. 4).

**Fig 5:**
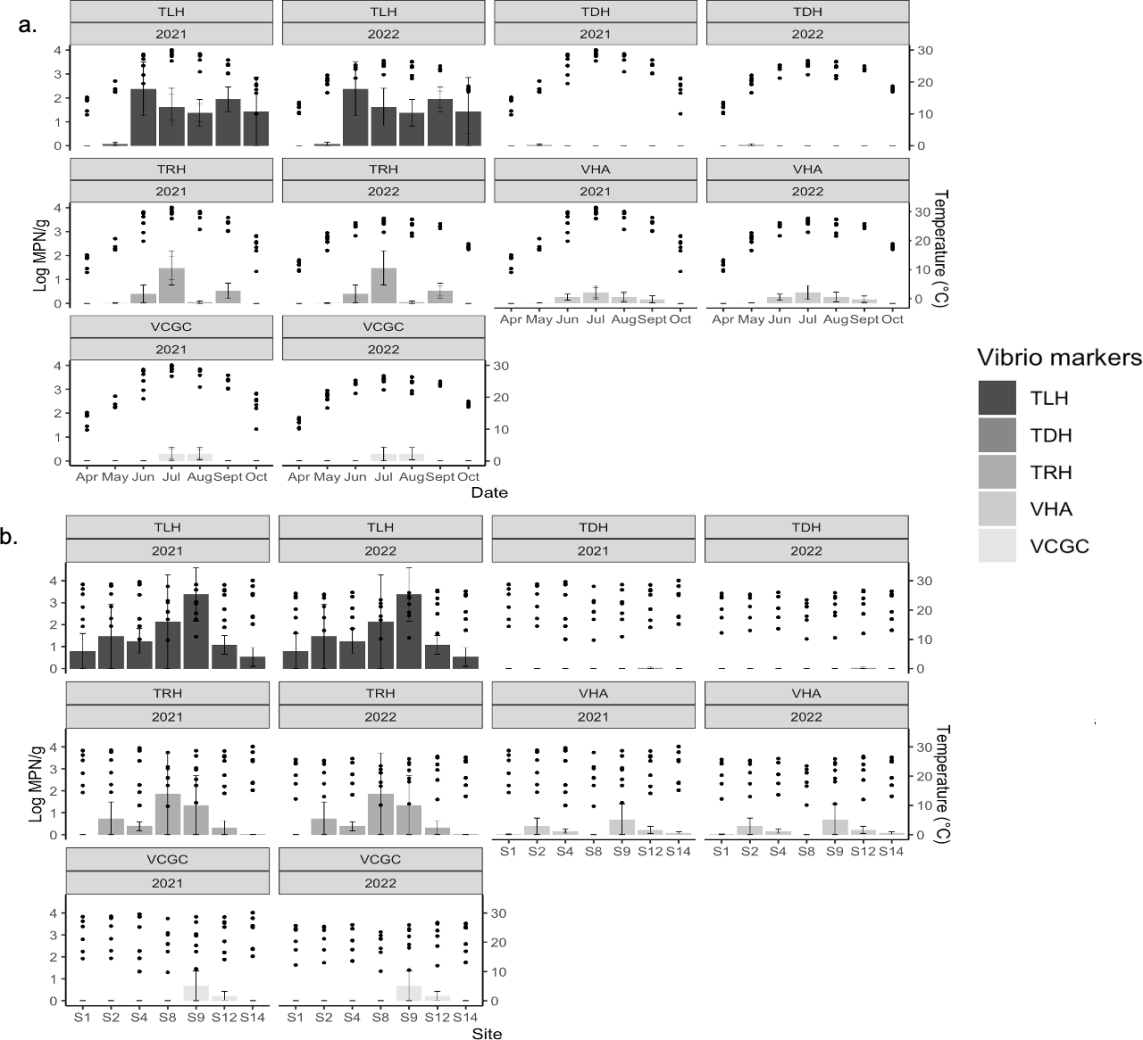
Temperature distribution overlaid with *Vibrio* spp. occurrence in *Mnemiopsis leidyi* tissues collected from the Maryland Coastal Bays in 2021–2022. *Vibrio* are represented as *V. parahaemolyticus* (*tlh*), *V. vulnificus* (*vvhA*), pathogenic *V. parahaemolyticus* (*tdh*^+^ and *trh*^+^), and pathogenic *V. vulnificus* (*vcgC*^+^). (**a**) Means and standard errors for *Vibrio* genes within months, *n* = 43. (**b**) Means and standard errors for *Vibrio* genes within sites, *n* = 43.

The *tlh*^+^ *V. parahaemolyticus* gene was detected in homogenized *M. leidyi* tissues from May to October, with the highest concentrations detected in June (Fig. 5a) and at all sites, with the highest concentrations occurring at site S9 (Fig. 5b). The *tdh*^+^ *V. parahaemolyticus* gene was not detected in any months or sites, but the *trh*^+^ gene was detected from June to September at sites S2, S4, S8, S9, and S12 with the highest concentrations occurring in June and site S8 (Fig. 5).

### *Vibrio vulnificus* prevalence in water and *Mnemiopsis leidyi* tissues

The concentrations and detection percentage of *V. vulnificus* in water samples and *M. leidyi* tissues using the species-specific and pathogenic gene targets are listed in Table 5. The *V. vulnificus* hemolysin A gene (*vvhA*) was detected in 20% of water samples and ranged in concentrations from 0.02 to 1.40 log MPN mL^−1^. The *vvhA* marker was found in 33% of homogenized *M. leidyi* tissue samples with concentrations ranging from 0.05 to 2.11 log MPN g^−1^. Using the *Vibrio* virulence correlated clinical gene (*vcgC*^*+*^), *V. vulnificus* was detected in 1% of water samples with a concentration ranging from 0.00 to 0.03 log MPN mL^−1^. The *vcgC*^+^ gene was detected in 8% of *M. leidyi* tissue samples with a concentration ranging from 0.30 to 2.04 log MPN g^−1^. There was a significant difference in total (*vvhA*) *V. vulnificus* found between years with higher concentrations occurring in 2021 (*P* < 0.05; Table 6).

The *vvhA V. vulnificus* gene was detected in water samples collected in June, July, August, and October, with the highest concentrations occurring in August (Fig. 4a). The *vvhA V. vulnificus* gene was detected in water samples from all sites, except S14. The highest *vvhA V. vulnificus* gene concentrations were detected at site S12 (Fig. 4b). The *vcgC^+^ V. vulnificus* gene was not detected in water samples collected in any month or site (Fig. 4).

The *vvhA V. vulnificus* gene was detected in homogenized *M. leidyi* tissues from June to September, with the highest concentrations detected in July (Fig. 5a). The *vvhA V. vulnificus* marker was detected in *M. leidyi* tissues from all sites, except S8, with the highest concentrations occurring at S9 (Fig. 5b). The *vcgC*^+^ *V. vulnificus* gene was detected in *M. leidyi* tissues in July and August, and only at sites S9 and S12 (Fig. 5).

### Correlation analysis of *Mnemiopsis leidyi, Vibrio*, harmful algae biomass, and abiotic parameter by season

**Fig 6:**
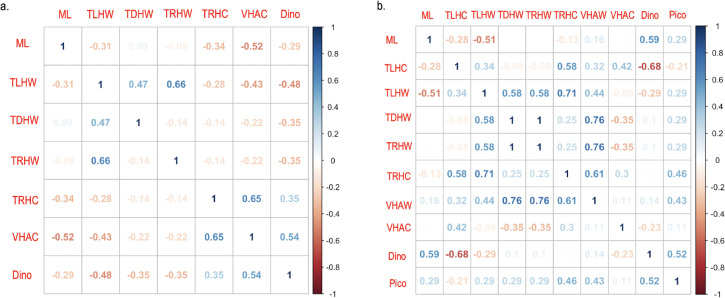
Seasonal correlation analysis between *Mnemiopsis leidyi*, *V. parahaemolyticus, V. vulnificus,* and *Vibrio* pathogenic markers, and dinoflagellate and picoplankton biomass collected from the Maryland Coastal Bays in 2021–2022. (**a**) Spring months from April to May. (**b**) Summer months from June to August. The blue-shaded scale indicates a positive relationship, and the red-shaded scale indicates a negative relationship. Abbreviation definitions: ML = *M. leidyi*, W = environmental sample, C = *M. leidyi* sample, Dino = dinoflagellate biomass, Pico = picoplankton biomass, and TLH, TDH, TRH, and VHA = *Vibrio*genetic markers.

Using the data collected in the spring, a correlation analysis of *M. leidyi* concentrations, *Vibrio* spp. concentrations, and harmful algae biomass revealed a positive association between the *V. vulnificus* gene marker *vvhA* in *M. leidyi* (VHAC) and total dinoflagellate biomass (Dino) (VHAC vs Dino) with a coefficient value >0.5 and *P* value of < 0.05. There was a negative association between *vvhA* in *M. leidyi* and *M. leidyi* concentrations (ML) (VHAC vs ML) (Fig. 6). Using data collected in the summer, a correlation analysis revealed a positive association among total dinoflagellate biomass, *M. leidyi* concentrations, and picoplankton biomass, (Dino vs ML) and (Dino vs Pico) with a coefficient value >0.5 and *P* value of < 0.05. There were no strong associations between harmful diatom biomass and any variable (Fig. S2).

**Fig 7:**
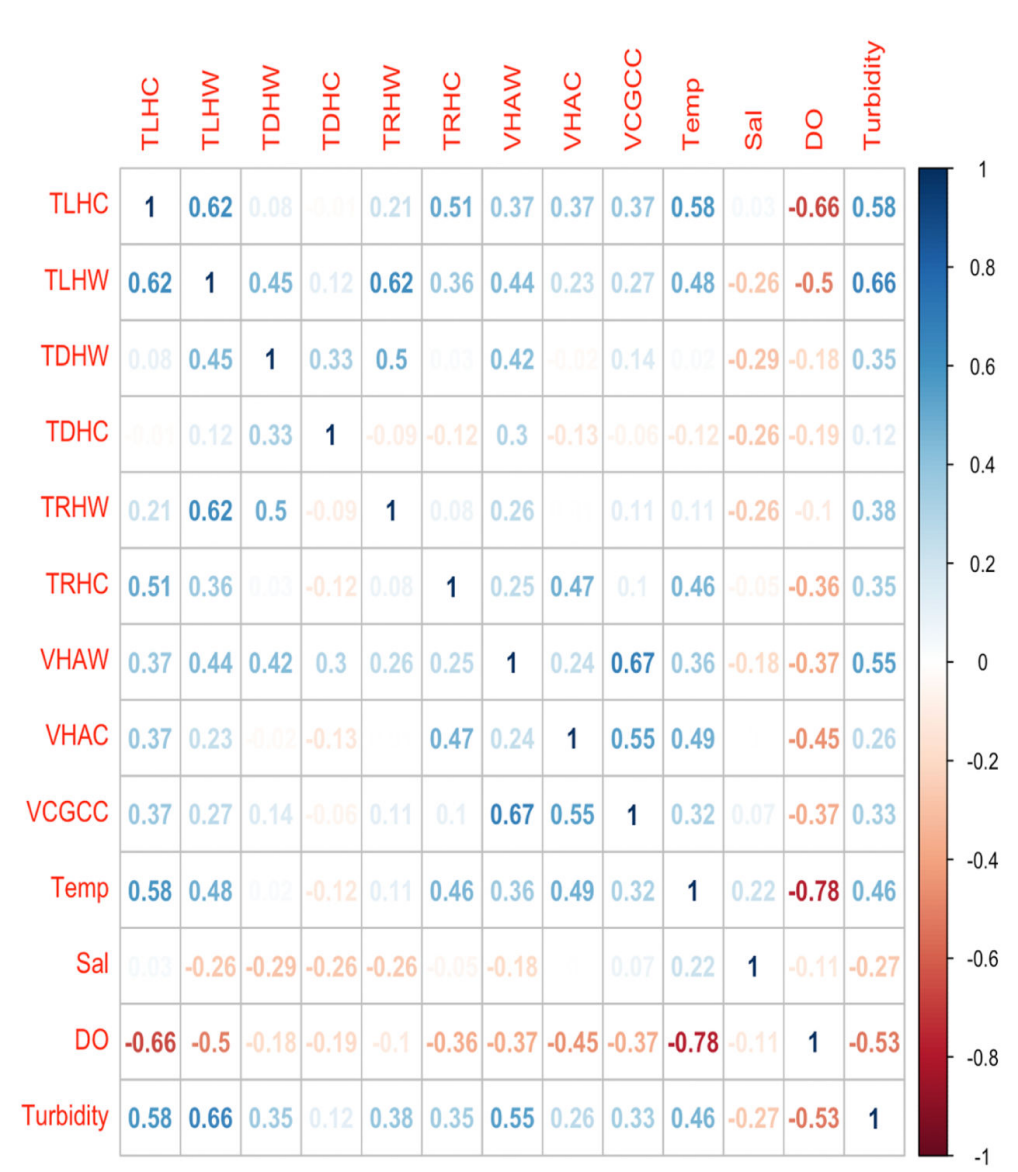
Correlation analysis between variables collected from the Maryland Coastal Bays in 2021–2022, including *V. parahaemolyticus*, *V. vulnificus,* and pathogenic *Vibrio* markers and environmental parameters. The blue-shaded scale indicates a positive relationship, and the red-shaded scale indicates a negative relationship. Abbreviation definitions: W = environmental sample, C = *Mnemiopsis leidyi* sample, Temp = temperature, Sal = salinity, DO = dissolved oxygen, and TLH, TDH, TRH, VHA, and VCG = *Vibrio*genetic markers.

For *V. parahaemolyticus* markers, *tlh V. parahaemolyticus* in water (TLHW) had a moderate positive correlation with *tlh V. parahaemolyticus* in *M. leidyi* (TLHC), *trh*^+^ *V. parahaemolyticus* in water (TRHW), and turbidity (TLHW vs TLHC), (TLHW vs TRHW), and (TLHW vs turbidity) (Fig. 7). Total *tlh V. parahaemolyticus* in *M. leidyi* had a positive correlation with *trh*^+^ *V. parahaemolyticus* in *M. leidyi* (TRHC), temperature (Temp), and turbidity (TLHC vs TRHC), (TLHC vs Temp), and (TLHC vs turbidity). However, there was a negative association between total *tlh V. parahaemolyticus* in *M. leidyi*, *tlh V. parahaemolyticus* in water, and dissolved oxygen levels (DO), (TLHC vs TLHW), and (TLHC vs DO). There was a moderate positive correlation with total *tdh*^+^ and *trh^+^ V. parahaemolyticus* in water (TDHW vs TRHW). For *V. vulnificus* markers, *vvhA V. vulnificus* in water had a positive association with *vcgC^+^ V. vulnificus* in *M. leidyi* (VCGCC) and turbidity (VHAW vs VCGCC) and (VHAW vs turbidity). The *vvhA V. vulnificus* marker in *M. leidyi* had a positive association with *vcgC^+^ V. vulnificus* in *M. leidyi* (VHAC vs VCGCC) (Fig. 7). All associations had a coefficient value >0.5 and *P* value of < 0.05.

### Modeling the relationship between *M. leidyi, Vibrio* spp., HABs, and environmental parameters

A GLM was used to determine which explanatory variables best described the detection of *Vibrio* spp. in *M. leidyi* and water samples. Temperature was the strongest determinant of total (*vvhA*) *V. vulnificus* in homogenized *M. leidyi* tissues (Table 1; Table S1), whereas turbidity exerted the most influence of total (*vvhA*) *V. vulnificus* in water (Table 2; Table S3). When temperature was combined with salinity and dissolved oxygen, model performance did not improve for (*vvhA*) *V. vulnificus*. Turbidity and temperature contributed the most in detecting total (*tlh*) and (*trh^+^*) *V. parahaemolyticus* in homogenized *M. leidyi* tissues (Table 1; Table S4). Turbidity also contributed the most in detecting total (*tlh*) and potentially pathogenic (*trh^+^*) and (*tdh^+^) V. parahaemolyticus* in water (Table 2; Table S3). For nutrients, TDN combined with NH_4_^+^ contributed the most in detecting (*trh*^+^) *V. parahaemolyticus* in water (Table 7; Table S5). The same GLM was used on *tdh*^+^ and *vcgC*^+^ in *M. leidyi* tissues, but the null model was the best fit in all scenarios. Inclusion of HAB data did not improve model explanatory powers.

**TABLE 7:** Model comparison of general linear models with *Trh ^+^* in the environment as the response variable

Model no	Explanatory variables	AICc^*a*^	Delta	Weight^*b*^	RMSE^*c*^
M4	*Trh* ~ TDN + NH4	24.0	0.00	0.605	0.323
M2	*Trh* ~ TDN + NH4 + NO2.NO3	26.9	2.90	0.142	0.394
M3	*Trh* ~ TDN + NO2.NO3	27.8	3.84	0.089	0.321
M1	*Trh* ~ TDN	27.9	3.95	0.084	0.350
Null	*Trh* ~ 1	28.0	4.04	0.080	0.373

^*a*^ AICc, Akaike’s information criterion. A lower AICc indicates a better model.

^*b*^ wt, weight.

^*c*^ RMSE, root-mean-square deviation.

A sensitivity analysis was performed in R Studio using base R and the sensitivity package to assess the reliability of the models and to help identify which model predictions were influenced by 10% changes in input variables, following Saltelli et al. (69). This analysis provided additional insight into the robustness of the model and highlights, through positive percent change values, that turbidity and temperature changes have the greatest impact on predicting the presence of *V. parahaemolyticus* and *V. vulnificus* in water and *M. leidyi* tissue samples across the spring and summer seasons (Table S3 through S5).

## DISCUSSION

### Environmental parameters and biota distribution

The MCBs can be partitioned into the northern (Assawoman [sites S1 and S2] and Isle of Wight [site S4]) and southern (Sinepuxent [site S9], Newport [site S12], and Chincoteague [site S14] Bays) regions, divided by Ocean City Inlet (site S8). Most of the inflow in the northern bays is freshwater discharge from the St. Martin River, while the southern bays receive most of their inflow via Ocean City Inlet (11). Elevated nutrients and a long residence time influence the water quality throughout the MCBs (11, 61). Nutrient concentrations measured between 2021 and 2022 were within the range of previously reported observations (61, 70, 14). Of note, high mean concentrations of PO_4-_ and TDP were observed in May 2022, which was likely due to an extensive rain event that occurred two or three days prior to sampling (https://waterdata.usgs.gov/md/nwis/rt). Seasonal mean nitrogen concentrations were consistent throughout both years. Nutrient concentrations varied little within sites, but consistently higher concentrations of NH_4_^+^, NO_3_:NO_2_^-^, and TDN were found in Assawoman and Isle of Wight Bays compared to other locations, likely due to the influence of urban runoff and treated effluents from the Ocean Pines wastewater treatment plant (70, 71).

There was no significant difference in HAB concentrations between sites, which means that, when present, the same HAB species occurring at multiple sites had similar cell concentrations. However, HAB species composition varied spatially and temporally. The dinoflagellate *K. veneficum* was the most abundant HAB species in Chincoteague Bay, consistent with observations made by Oseji et al. (37). The HAB community in the northern MCBs was dominated by the dinoflagellate *P. minimum*, particularly in spring and early summer, consistent with earlier regional studies (13, 14). Recent studies by Kim et al. (34) and Martinez-Mercado et al. (72), have shown an association between bacteria, including *Vibrio* spp., and *Prorocentrum* spp. during blooms and speculate that this is due to bacteria-mediated mineralization of phosphorus or some other metabolic function. Nitrogen constituents are a major driver of HABs in lagoonal systems (27, 73, 74), but because concentrations were relatively consistent during this study, we did not observe a distinct relationship between nitrogen and HABs.

*M. leidyi* was present in both years and at all sites, but there was a significant difference in its abundance between sites. *M. leidyi* was most abundant in Isle of Wight Bay and Newport Bay, northern and southern sites that are heavily influenced by freshwater inputs from the St. Martin River and a series of creeks, respectively (11, 75). These sites had average turbidity levels >11.5 PSU, water temperatures >21°C, and salinities <30, all median values compared to other sites in this study. The TDP concentrations (0.04 µM) were low compared to values ranging from 0.06 µM to 0.50 µM at other sites. TDN was the most abundant class of nitrogen measured at these sites, albeit nitrogen concentrations were lower here than other sites in the study. It is well known that *M. leidyi* population growth and reproduction are constrained by temperature and salinity (76, 77), but the semi-enclosed construct of Newport and Isle of Wight Bays may provide the habitat necessary to allow *M. leidyi* to grow (average concentration of 947 organisms per m^3^), as has been documented in other locations where this species has invaded coastal systems with concentrations ranging from 500 to 940 organisms per m^3^ (78, 79).

Using genetic markers, it was determined that both *V. parahaemolyticus* and *V. vulnificus* were present at all sites in 2021 and 2022. *Vibrio* concentrations detected in water samples and in *M. leidyi* tissues varied throughout the study, but there was no significant difference among sites. These findings coincide with previous reports on *Vibrio* spp. and *M. leidyi* distribution in the MCBs (5, 80, 81). There was a significant difference in the concentration of total *tlh*^+^ *V. parahaemolyticus* in water samples between years and months, with greatest concentrations occurring from July to September 2021. We also observed a significant difference in *vvhA V. vulnificus* in water between years, with more *V. vulnificus* being detected in 2021 than 2022. These findings could be due to differences in salinity, which was significantly lower in 2021 compared to 2022. The average salinity was 27 ppt in 2021 (range from 20 to 32 ppt) and 29 in 2022 (range 19–36 ppt). The salinity range in 2021 fell within that reported by Smalls et al. (81) for optimal for *V. vulnificus* growth in the MCB, but the 2022 salinity range exceeded this optimum. However, a relationship between *V. parahaemolyticus* and salinity is less straightforward (82, 83), and Davis et al. (84) have indicated that salinities between 10 and 23 ppt are more favorable for this species in the mid-Atlantic region. Warmer water temperatures can also influence the abundance of *V. parahaemolyticus* and *V. vulnificus* (85–87). The average sea water temperatures in the MCBs were higher in 2021 compared to 2022. The highest temperatures were recorded from July to September 2021, ranging from 21.1°C to 30.0°C, making conditions suitable for *V. parahaemolyticus* and *V. vulnificus* to thrive.

### Correlation analyses

A correlation analysis of data collected in the spring revealed a positive association between the *V. vulnificus* marker *vvhA* in *M. leidyi* and total dinoflagellate biomass. Previous studies have shown that organic carbon, nitrogen, and phytoplankton blooms have been found to influence *V. vulnificus* abundance in water and in filter feeders (88, 89). *Vibrio* spp. have been found on a broad spectrum of planktonic hosts >20 µm, including copepods, ciliates, cnidarians, tunicates, diatoms, and dinoflagellates (88). Therefore, it is not surprising to find an association among dinoflagellates, *Vibrio*, and *M. leidyi*. However, we found a negative association between *vvhA* in *M. leidyi* tissues and *M. leidyi* concentrations, likely because *M. leidyi* abundance is influenced by temperature and prey concentrations (76, 90, 91), both of which are reduced in the cooler waters of spring. In this study, *M. leidyi* abundance was higher in summer in comparison to spring. Picoplankton was not observed in abundant concentrations in the spring and therefore was not added in the spring correlation analysis.

A correlation analysis of summer data revealed a positive association between dinoflagellate biomass and *M. leidyi* concentrations, as well as between dinoflagellates and picoplankton biomass. This observation has been found in other estuaries, and it is likely due to *M. leidyi* exerting predation pressure on mesoplankton, which subsequently influences dinoflagellate and picoplankton abundance (92, 93). In this study, the picoplankton category was composed of phototrophic cells between 2 and 3 µm in size. Within this category, we identified both picocyanobacteria and the brown tide organism *A. anophagefferens*. Co-occurrence of cyanobacteria and dinoflagellate blooms is often due to nutrients made available by the cyanobacterial fraction of the phytoplankton population (94, 95). McNamara et al. (93) demonstrated that *A. anophagefferens* enhances the feeding of larval *M. leidyi*. We did not observe a strong correlation between *M. leidyi* and picoplankton, likely due to the monthly sampling scheme that failed to capture the full dynamics of picoplankton blooms (noted as changing daily to weekly in shallow, lagoonal systems by (73) in the MCBs and/or our focus on examining adult *M. leidyi*. We also observed a negative association between *tlh V. parahaemolyticus* in *M. leidyi* tissues and dinoflagellate biomass during the summer months. This may be due to reduced *V. parahaemolyticus* populations during dinoflagellate blooms (10^7^–10^10^ dinoflagellate cells L^−1^) (14, 96) or the feeding preferences of *M. leidyi*, which is known to be size-specific and influenced by its own biovolume and water temperature (97, 98). We found no associations between diatom biomass and any variable. This may be due to our limited focus on harmful diatom taxa and not the entire diatom community. Including a broader assessment of the phytoplankton community may provide more insights into the dynamics between HABs, *M. leidyi*, and *Vibrio* spp. and should be considered in future studies.

Temperature and turbidity were two environmental factors that had a positive association with total *tlh V. parahaemolyticus* found in *M. leidyi* tissues. This is not surprising as high densities of *V. parahaemolyticus* in oysters have been positively correlated with seawater temperatures (19, 87). In the Chesapeake Bay, a positive association has been found between turbidity, oysters, and *V. parahaemolyticus* (84, 99). Higher nutrient levels are often associated with higher turbidity, fecal matter, and phytoplankton matter and may stimulate *V. parahaemolyticus* growth while also providing substrates for *Vibrio* that are a size more easily taken up by filter feeders (21, 83, 99). Dissolved oxygen is also another variable that has been widely studied in relation to *V. parahaemolyticus*. Davis et al. (84) and Hartwick et al. (100) found a negative association between dissolved oxygen and total (*tlh) V. parahaemolyticus*. This coincides with our findings wherein total (*tlh) V. parahaemolyticus* in *M. leidyi* had a negative association with dissolved oxygen, likely due to the inverse relationship between dissolved oxygen and water temperature (101).

There is a lack of studies describing the interaction between *Vibrio* spp. and *M. leidyi*. Concentrations of *Vibrio* spp. in *M. leidyi* were found to be comparable to concentrations found in oysters (18, 102, 103). A previous study in the MCBs observed concentrations of *Vibrio* spp. in oysters ranging from 0 to 3.5 log MPN g^−1^ (18), and we found similar concentrations in *M. leidyi*. This is likely due to the similar feeding strategies of jellyfish and oysters, which prefer larger detrital particles and zooplankton (88, 104, 105). Since *Vibrio* spp. are known to attach to zooplankton, this association makes them more susceptible to ingestion by filter feeders (106).

### Ecological modeling

A GLM, with follow-up sensitivity analysis, showed that turbidity contributed the most in detecting total *tlh*, *trh^+^*, *tdh ^+^*, and *vvhA* in the environment. This is consistent with recent studies in the Chesapeake Bay (84, 86). Turbidity is the resuspension of particles and sediments into the water column. These particles provide not only a substrate for *Vibrio* spp. to attach to, but also a way to reintroduce *Vibrio* spp. back into the water column as part of the microbial loop (107). In *M. leidyi* tissue samples, temperature contributed the most in detecting total *vvhA V. vulnificus*. Studies done in the Chesapeake Bay and the Gulf of Mexico found a similar trend between warmer temperatures and higher concentrations of *V. vulnificus* in oysters (108–110).

A combination of temperature and turbidity was determined as the best predictor for detecting total *tlh* and *trh^+^ V. parahaemolyticus* in homogenized *M. leidyi* tissues, similar to findings on *V. parahaemolyticus* abundance in oysters (19, 87). Turbidity and temperature together were found to be predictors for the presence of *V. parahaemolyticus* in water and oysters (83, 99, 111), similar to our findings within *M. leidyi* tissues and water.

Nutrients can also play a role in *Vibrio* abundance, and recent studies have found an association between nutrients and *Vibrio* concentrations in marine environments (27, 28, 112). In our study, the correlation analysis revealed a positive association between TDN and *trh*^+^ *V. parahaemolyticus* in water (Table 7). Bacteria are known to rapidly assimilate low-molecular-weight compounds, such as amino acids and urea (113), and *Vibrio* spp. can readily use different types of nitrogenous constituents (114). This highlights the importance of nutrient reduction plans that aim to improve water quality and safety of the MCBs and warrants investigation of field-deployable TDN sensors (described in [115]) that could provide near-real-time TDN data.

Even though *V. parahaemolyticus* and *V. vulnificus* were detected in *M. leidyi* tissues and water samples, there was no strong relationship between their abundances. The lack of association between *M. leidyi* and *Vibrio* abundances could be due to the relatively small sample size of this study or to other environmental factors that were not considered, such as the competing microbiome and the total phytoplankton community. These groups, plus *M. leidyi* and *Vibrio* populations, have been found to coincide with zooplankton assemblages (104, 106, 116), which in the MCB are tied to seasonal shifts in temperature and salinity (23). We chose to investigate the potential interaction between jellyfish, harmful algae, *Vibrio*, and discrete water quality parameters, as several water quality constituents and phytoplankton populations are increasingly being monitored for in this region using temporally and spatially rigorous remote sensing technologies (26, 117, 118). If it was possible to leverage data from these remote sensing technologies and augment existing *Vibrio* models (8, 26), resource managers would have more data available to them, besides *in situ* sample data only, to detect and track *Vibrio* populations. While this assessment showed some correlations between *Vibrio* spp. and parameters that could be remotely sensed, such as water temperature, salinity, and phytoplankton concentrations, future studies should consider a whole community approach and specifically phyto- and zooplankton interactions with *Vibrio* spp. and *M. leidyi* for better predictive and modeling powers.

Even though there was not a significant association between *M. leidyi* abundance and *Vibrio*, we did find a significant association between *M. leidyi* abundance and dinoflagellate biomass during the summer. Seasonal dynamics and HAB community composition can play a role in *M. leidyi* abundance and should be studied further. Our study provided new knowledge on the ecology of *Vibrio* spp., HABs, and *M. leidyi*. Temperature and turbidity were major factors influencing *M. leidyi* abundance in the MCBs. Sea surface temperatures and turbidity are increasing in the Chesapeake Bay region (61, 119), which may lead to an increase in *M. leidyi* abundance in local estuaries and an alteration in microbial and food web dynamics. This study provides novel information on the interactions of *M. leidyi*, *Vibrio* spp., and HABs in the MCBs. Moreover, data from this study can be used for future ecological models on HAB and *Vibrio* dynamics in estuarine systems.

## SUPPLEMENTAL MATERIAL

Fig. S1 spectrum.00978-25-s0001.tif Relative abundance of harmful algal bloom species, by site, in the Maryland Coastal Bays from 2021 to 2022.

Fig. S2 spectrum.00978-25-s0002.tif Seasonal correlation analysis between *M. leidyi*, *V. parahaemolyticus, V. vulnificus, and Vibrio spp. pathogenic markers and diatom biomass (HAB species only) examined in MCBs in 2021-2022.*

Supplemental material spectrum.00978-25-s0003.docx Tables S1 to S5; Supplemental figure captions.

ASM does not own the copyrights to Supplemental Material that may be linked to, or accessed through, an article. The authors have granted ASM a non-exclusive, world-wide license to publish the Supplemental Material files. Please contact the corresponding author directly for reuse.
