# Supplementary figures and images for: Investigating the relationship between the comb jellyfish, Mnemiopsis leidyi, and the abundance of pathogenic Vibrio spp. and harmful algae species in the Maryland Coastal Bays
Source: Microbiol Spectr. 2025 Dec 30;14(2):e00978-25. doi: 10.1128/spectrum.00978-25 (PMC12889146; doi:10.1128/spectrum.00978-25)

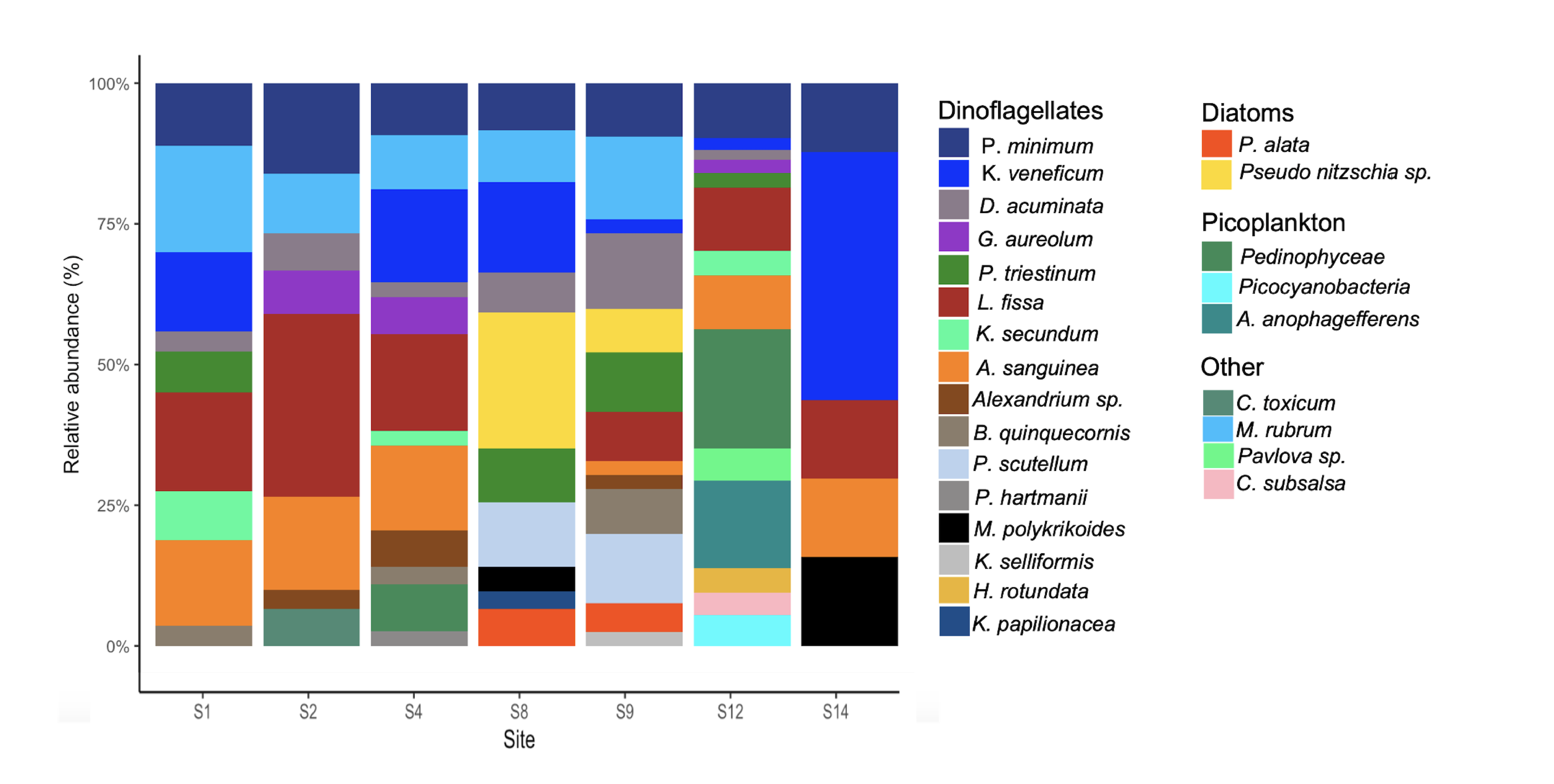

Supplement: Fig. S1 — Relative abundance of harmful algal bloom species, by site, in the Maryland Coastal Bays from 2021 to 2022. [file spectrum.00978-25-s0001.tif]

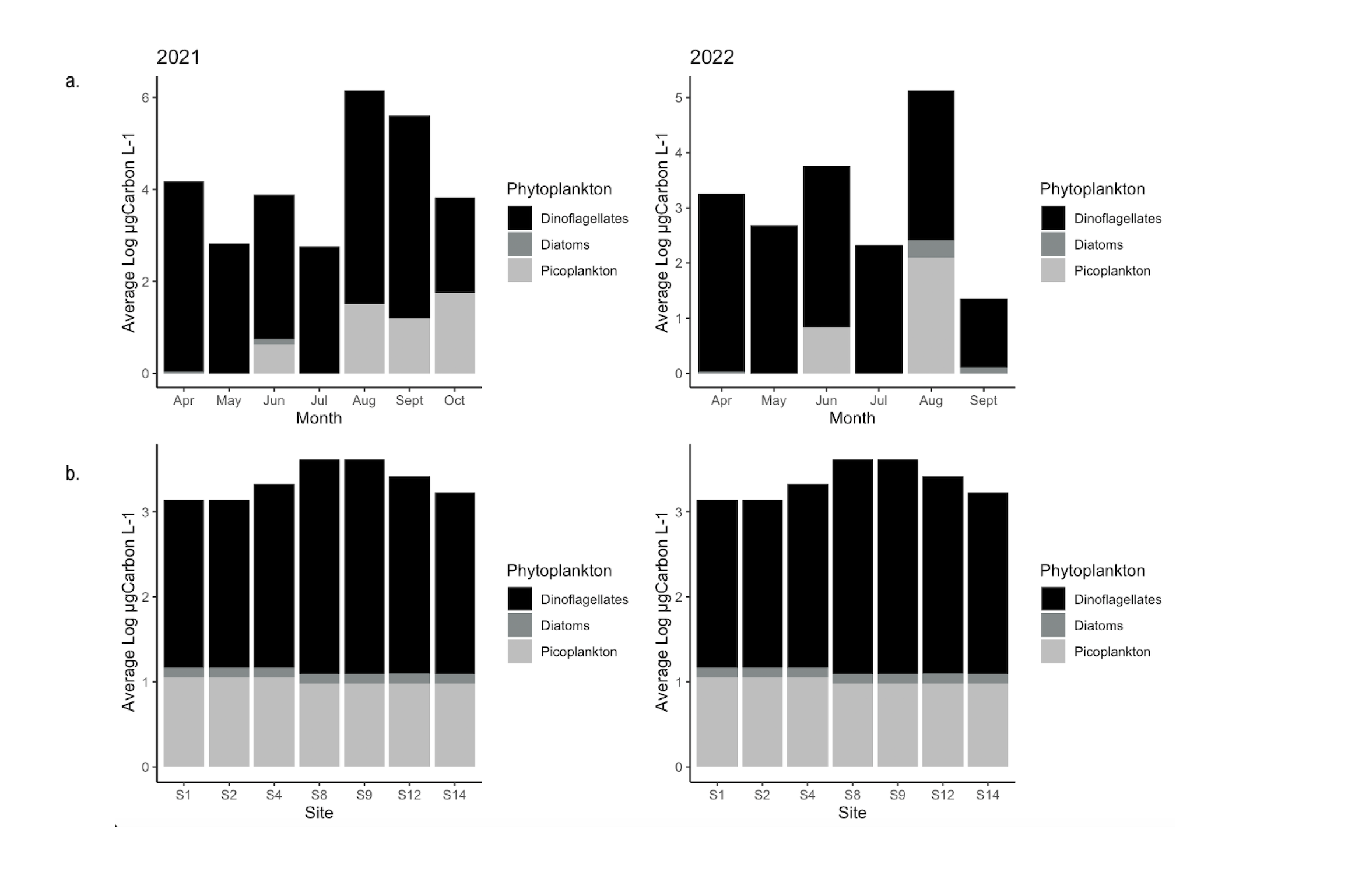

Supplement: Fig. S2 — Seasonal correlation analysis between M. leidyi, V. parahaemolyticus, V. vulnificus, and Vibrio spp. pathogenic markers and diatom biomass (HAB species only) examined in MCBs in 2021-2022. [file spectrum.00978-25-s0002.tif]
