# Supplementary material for: Investigating the relationship between the comb jellyfish, Mnemiopsis leidyi, and the abundance of pathogenic Vibrio spp. and harmful algae species in the Maryland Coastal Bays
Source: Microbiol Spectr. 2025 Dec 30;14(2):e00978-25. doi: 10.1128/spectrum.00978-25 (PMC12889146; doi:10.1128/spectrum.00978-25)
Supplement: Supplemental material — Tables S1 to S5; Supplemental figure captions. [file spectrum.00978-25-s0003.docx]

**Supplemental Section**

**FIGURE 1S.** Relative abundance of harmful algal bloom species, by site, in the Maryland Coastal Bays from 2021 – 2022.

**FIGURE 2S**: Seasonal correlation analysis between *M. leidyi, V. parahaemolyticus,* *V.vulnificus* and *Vibrio* spp. pathogenic markers and diatom biomass (HAB species only) examined in MCBs in 2021-2022. a) Spring months from April-May. b) Summer months from June-August. The blue-shaded scale indicates a positive relationship, and the red-shaded scale indicates negative relationship. Abbreviation definition are ML= *M. leidyi*, W= environments sample and C = *M. leidyi* sample.

**SUPPLEMENTAL TABLE 1.** Comparison of environmental metrics (P values) in the Maryland Coastal Bays from 2021 and 2022.

| Maryland Coastal Bays | |
| --- | --- |
| 2021-2022 | P value |
| Dinoflagellate^a^ | **6.51 × 10^-5^** |
| Picocyanobacteria^a^ | **0.005** |
| Diatoms^a^ | **0.019** |
| *M. leidyi*^b^ | 0.133 |
| Temperature ^c^ | 0.21 |
| Salinity ^d^ | **5.6 × 10^-3^** |
| Dissolved oxygen ^e^ | 0.86 |
| Turbidity ^f^ | 0.10 |
| 2021-2022 | P value |
| NH_4_^+ g^ | 0.35 |
| NO_3_^-^:NO_2_^-^ ^g^ | 0.21 |
| TDN ^g^ | 0.64 |
| PO_4_^3-g^ | **1.5 × 10^-2^** |
| TDP^g^ | **1.7 × 10^-2^** |
| ^a^ Biomass μg Carbon L^-1^, ^b^Density mL^-1^; ^c^ °C; ^d^ psu; ^e^mg L^-1^, ^f^NTU, ^g^μM,  P values were determined by a one-way ANOVA.  For 2021 sample size (Water = 49 and *M. leidyi* = 30). For 2022 sample size ( Water = 46 and *M. leidyi* = 13). | |

**SUPPLEMENTAL TABLE 2.** Complete list of HAB taxa identified during the *Vibrio* – *Mnemiopsis leidyi* study conducted between 2021 and 2022.

| **Algal Category** | **Taxa** | **Harmful Impacts** | **Reference** |
| --- | --- | --- | --- |
| **Diatom** | *Proboscia alata* | High biomass blooms | Thomas et al. (2014) |
|  | *Pseudo-nitzschia* spp. | Potential domoic acid producer | Lassus et al. (2016) |
| **Dinoflagellate** | *Akashiwo sanguinea* | High biomass blooms, finfish and shellfish mortalities | Lassus et al. (2016) |
|  | *Alexandrium* sp. | Potential saxitoxin producer | Lassus et al. (2016) |
|  | *Blixaea quinquecornis* | High biomass blooms | Rodriguez-Gómez et al. (2019) |
|  | *Dinophysis acuminata* | Okadaic acid and dinophysistoxin producer | Lassus et al. (2016) |
|  | *Gonyaulax spinifera* | Potential yessotoxin producer | Lassus et al. (2016) |
|  | *Gymnodinium aureolum* | High biomass blooms | Lassus et al. (2016) |
|  | *Gyrodinium spirale* | High biomass blooms | Vila and Masó et al. (2005) |
|  | *Heterocapsa rotundata* | High biomass blooms | Lassus et al. (2016) |
|  | *Karenia papilionacea* | High biomass blooms, brevetoxin producer | Lassus et al. (2016) |
|  | *Karenia selliformis* | High biomass blooms, gymnodimine producer | Lassus et al. (2016) |
|  | *Karlodinium veneficum* | Karlotoxin producer | Lassus et al. (2016) |
|  | *Kryptoperidinium secundum* | High biomass blooms | Tillman et al. (2025) |
|  | *Levanderina fissa* | High biomass blooms | Lassus et al. (2016) |
|  | *Margalefidinium polykrikoides* | High biomass blooms | Lassus et al. (2016) |
|  | *Pheopolykrikos hartmannii* | Uncharacterized ichthyotoxins | Lassus et al. (2016) |
|  | *Prorocentrum micans* | High biomass blooms | Cruz-Balladare et al. (2023) |
|  | *Prorocentrum minimum* | High biomass blooms | Lassus et al. (2016) |
|  | *Prorocentrum scutellum* | High biomass blooms | Taş et al. (2016) |
|  | *Prorocentrum triestinum* | High biomass blooms | Cruz-Balladare et al. (2023) |
| **Picoplankton** | *Aureococcus anophagefferens* | High biomass blooms | Gobler and Sunda (2012) |
|  | Pedinophyceae | High biomass blooms | Phlips et al. (2015) |
|  | unidentified picocyanobacteria | High biomass blooms | Śliwińska-Wilczewska et al. (2018) |
|  |  |  |  |
| Cruz-Balladares, V., Avalos, V., Vera-Villalobos, H., Cameron, H., Gonzalez, L., Leyton, Y., Riquelme, C. 2023. Identification of a *Shewanella halifaxensis s*train with algicidal effects on red tide dinoflagellate *Prorocentrum triestinum* in culture. *Marine Drugs* 21: 501. | | | |
| Gobler, C.J., Sunda, W.G. 2012. Ecosystem disruptive algal blooms of the brown tide species, *Aureococcus anophagefferens* and *Aureoumbra lagunensis*. *Harmful Algae* 14: 36-45. | | | |
| Lassus, P., Chaumérat, N., Hess, P., Nézan, E. and Reguera, B., 2016. Toxic and harmful microalgae of the world ocean. 523p. | | | |
| Phlips, E.J., Badylak, S., Lasi, M.A., Chamberlain, R., Green, W.C., Hall, L.M., Hart, J.A., Lockwood, J.C., Miller, J.D., Morris, L.J., Steward, J.S. 2015. From red tides to green and brown tides: bloom dynamics in a restricted subtropical lagoon under shifting climatic conditions. *Estuaries and Coasts* 38: 886-904. | | | |
| Rodríguez-Gómez, C.F., Vázquez, G., Aké-Castillo, J.A., Band-Schmidt, C.J., Moreno-Casasola, P. 2019. Physicochemical factors related to Peridinium quadridentatum (F. Stein) Hansen (Dinophyceae) blooms and their effect on phytoplankton in Veracruz, Mexico. *Estuarine, Coastal and Shelf Science*230: 106412. | | | |
| Śliwińska-Wilczewska S, Maculewicz J, Barreiro Felpeto A, Latała A. 2018. Allelopathic and bloom-forming picocyanobacteria in a changing world. *Toxins* 10: 48. doi.org/10.3390/toxins10010048 | | | |
| Taş, S., Ergül, H.A., Balkis, N. 2016. Harmful algal blooms (HABs) and mucilage formations in the Sea of Marmara. In: Özsoy, E., Çağatay, N.M., Balkis, N., Balkis, N., Öztürk, B. (Eds.). The Sea of Marmara: Marine Biodiversity, Fisheries, Conservation and Governance. Turkish Marine Research Foundation, Publication No. 42. pp. 768-786. | | | |
| Thomas, A.M., Sanilkumar, M.G., Vijayalakshmi, K.C., Hatha, A.M., Saramma, A.V. 2014. Proboscia alata (Brightwell) Sandström bloom in the coastal waters off Bekal, southwest India. *Current Science* 106: 1643-1646. | | | |
| Tillmann, U. Gottschling, M., Wietkamp, S., Peeken, I., Wolny, J., Yamada, N. 2025. Diversity of *Kryptoperidnium* (Peridiniales, Dinophyceae): Morphological description and molecular phylogenetics of *Kryptoperidinium secundum* sp. nov. *Protist*: doi.org/10.1016.j.protis.2025.126120 | | | |
| Vila, M. and Masó, M., 2005. Phytoplankton functional groups and harmful algae species in anthropogenically impacted waters of the NW Mediterranean Sea. *Scientia Marina*, 69(1): 31-45. | | | |

| ***vvhA*** | | | | | |
| --- | --- | --- | --- | --- | --- |
| **Variable** | **M3 (β / ΔPred)** | **M4(β / ΔPred)** | **M5(β / ΔPred)** | **M6(β / ΔPred)** | **M7(β / ΔPred)** |
| Turbidity | 1.8 E-02/ 18% | 1.8 E-02/ 13% | 1.8 E-02/ 13% | 1.8 E-02/ 12% | 1.8 E-02/ 42% |
| Temperature | 3.5 E-03/ -5% | 4.7 E -05 / -.4% | NA | -2.0 E-03/ -.9 % | NA |
| Salinity | -3.7 E-03/ -5% | -5.4 E -03/ -3% | -5.7 E -03/ -3 % | NA | NA |
| DO | 2.0 E-02/ 7% | NA | NA | NA | NA |
| ***Tlh*** | | | | | |
| **Variable** | **M3 (β / ΔPred)** | **M4(β / ΔPred)** | **M5(β / ΔPred)** | **M6(β / ΔPred)** | **M7(β / ΔPred)** |
| Turbidity | 4.6 E-02/7 % | 4.6 E -02 /7% | 5.9 E-02/14% | 5.6 E-02/12 % | 6.0 E-02/22 % |
| Temperature | 4.7 E-02/14 % | 4.5 E -02 /14% | NA | 2.3 E-02/4 % | NA |
| Salinity | -7.0 E-02/-23% | -7.1 E-02 /-25% | -4.1 E-02/-25% | NA | NA |
| DO | 1.1 E-02/1% | NA | NA | NA | NA |
| ***Trh*** | | | | | |
| Variable | **M3 (β / ΔPred)** | **M4(β / ΔPred)** | **M5(β / ΔPred)** | **M6(β / ΔPred)** | **M7(β / ΔPred)** |
| Turbidity | 2.6 E-02/-12 % | 2.5 E-02/14 % | 2.8 E-02/10% | 3.1 E-02/-187% | 3.1 E-02/6 % |
| Temperature | 1.8 E-02/-72 % | 7.6 E-03/8% | NA | -3.0 E-03/54 % | NA |
| Salinity | -3.0 E-02/163% | -3.5 E-02/-58 % | -3.1 E-03/-7% | NA | NA |
| DO | 5.4 E-02/-98 % | NA | NA | NA | NA |
| ***Tdh*** | | | | | |
| Variable | **M3 (β / ΔPred)** | **M4(β / ΔPred)** | **M5(β / ΔPred)** | **M6(β / ΔPred)** | **M7(β / ΔPred)** |
| Turbidity | 6.0 E-03/52 % | 5.0 E-03/32 % | 4.7 E-03/63 % | 4.5 E-03/18 % | 4.3 E-03/162% |
| Temperature | -2.0 E-03/-60 % | -2.0 E-03/-43 % | NA | -5.0 E-4/-3 % | NA |
| Salinity | 7.0 E-03/298% | 6.0 E03/122% | 4.6 E-03/214% | NA | NA |
| DO | 4.0 E-03/40% | NA | NA | NA | NA |
| β = estimate coefficient, ΔPred **=** % change in prediction for a 10% increase in variable | | | | | |

**SUPPLEMENTAL TABLE 3**. Sensitivity analysis of general linear models with Vibrio spp. markers in environmental samples as the response variable.

**SUPPLEMENTAL TABLE 4.** Sensitivity analysis of general linear models with Vibrio spp. markers in *Mnemiopsis leidyi* tissue as the response variable.

| ***vvhA*** | | | | |  |  |
| --- | --- | --- | --- | --- | --- | --- |
| **Variable** | **M7 (β / ΔPred)** | **M8 (β / ΔPred)** | **M9 (β / ΔPred)** | **M10 (β / ΔPred)** | |  |
| Turbidity | NA | -2.7 E-02/8% | NA | NA |  |  |
| Temperature | 4.3 E-02/-13% | 3.1 E-02/-25% | 2.1 E-02/-285 % | 4.3 E-02/-9% |  |  |
| Salinity | NA | -2.8 E-02/27 % | -7.1 E-03/138 % | -2.1 E03/1% |  |  |
| DO | NA | -9.0 E-02/51% | -6.8 E-02/269% | NA |  |  |
| ***Tlh*** | | | | | | |
| **Variable** | **M3 (β / ΔPred)** | **M4(β / ΔPred)** | **M5(β / ΔPred)** | **M6(β / ΔPred)** | **M7(β / ΔPred)** | |
| Turbidity | 1.9 E-02/1 % | 3.5 E-02/3% | 7.9 E-02/7% | 3.0 E-02/2% | 7.0 E-02/5% | |
| Temperature | -1.9 E-02/-5% | 1.4 E-01/28% | NA | 1.5 E-01/29% | NA | |
| Salinity | -5.1 E-02/-16% | 3.3 E-02/9% | 1.3 E-01/32% | NA | NA | |
| DO | -8.1 E-01/-60% | NA | NA | NA | NA | |
| ***Trh*** | | | | | | |
| Variable | **M3 (β / ΔPred)** | **M4(β / ΔPred)** | **M5(β / ΔPred)** | **M6(β / ΔPred)** | **M7(β / ΔPred)** | |
| Turbidity | -1.4E-02/1% | -4.6 E-03/-1% | 3.3 E-02/6% | 9.3 E-02/0.1% | 3.1 E-01 /5% | |
| Temperature | 3.3 E-02/-29% | 1.2 E-01/65% | NA | 1.1 E-01/-3.2% | NA | |
| Salinity | -9.7 E-02/150% | -5.0 E-02/-28% | 3.3 E-02/17% | NA | NA | |
| DO | -4.6 E-01/198% | NA | NA | NA | NA | |
| β = estimate coefficient, ΔPred **=** % change in prediction for a 10% increase in variable | | | | | | |

**SUPPLEMENTAL TABLE 5**. Sensitivity analysis of general linear models with *Trh^+^* in the environment as the response variable.

| ***Trh*** | | | | | | | |
| --- | --- | --- | --- | --- | --- | --- | --- |
| Variable | **M1(β / ΔPred)** | | **M2(β / ΔPred)** | | **M3(β / ΔPred)** | | **M4(β / ΔPred)** |
| TDN | 5.8E-01/26% | 1.5 E00/25% | | 8.5 E-01/7% | | 1.4E 00/18 % | |
| NH4 | NA | -1.0 E01/-5% | | NA | | -7.9 E00/-2.7 % | |
| NO2.NO3 | NA | 5.4 E00/1% | | -1.1 E01/4 % | | NA | |
| β = estimate coefficient, ΔPred **=** % change in prediction for a 10% increase in variable | | | | | | | |

Reference:

1. Cruz-Balladares, V., Avalos, V., Vera-Villalobos, H., Cameron, H., Gonzalez, L., Leyton, Y. and Riquelme, C., 2023. Identification of a Shewanella halifaxensis Strain with Algicidal Effects on Red Tide Dinoflagellate Prorocentrum triestinum in Culture. Marine Drugs, 21(9), p.501.
2. Lassus, P., Chaumérat, N., Hess, P., Nézan, E. and Reguera, B., 2016. Toxic and harmful microalgae of the world ocean. 523p.
3. Phlips, E.J., Badylak, S., Lasi, M.A., Chamberlain, R., Green, W.C., Hall, L.M., Hart, J.A., Lockwood, J.C., Miller, J.D., Morris, L.J. and Steward, J.S., 2015. From red tides to green and brown tides: bloom dynamics in a restricted subtropical lagoon under shifting climatic conditions. Estuaries and Coasts 38: 886-904.
4. Rodríguez-Gómez, C.F., Vázquez, G., Aké-Castillo, J.A., Band-Schmidt, C.J., Moreno-Casasola, P. 2019. Physicochemical factors related to Peridinium quadridentatum (F. Stein) Hansen (Dinophyceae) blooms and their effect on phytoplankton in Veracruz, Mexico. *Estuarine, Coastal and Shelf Science*230: 106412.
5. Śliwińska-Wilczewska S, Maculewicz J, Barreiro Felpeto A, Latała A. 2018. Allelopathic and bloom-forming picocyanobacteria in a changing world. Toxins 10: 48. <https://doi.org/10.3390/toxins10010048>
6. Taş, S., Ergül, H.A. and Balkis, N., 2016. Harmful algal blooms (HABs) and mucilage formations in the Sea of Marmara. The Sea of, 768.
7. Thomas, A.M., Sanilkumar, M.G., Vijayalakshmi, K.C., Hatha, A.M. and Saramma, A.V., 2014. Proboscia alata (Brightwell) Sandström bloom in the coastal waters off Bekal, southwest India. Current science, 106(12), pp.1643-1646.
8. Vila, M. and Masó, M., 2005. Phytoplankton functional groups and harmful algae species in anthropogenically impacted waters of the NW Mediterranean Sea. Scientia Marina, 69(1), pp.31-45.
